# CSF-1/CSF-1R axis is associated with epithelial/mesenchymal hybrid phenotype in epithelial-like inflammatory breast cancer

**DOI:** 10.1038/s41598-018-27409-x

**Published:** 2018-06-21

**Authors:** Kazuharu Kai, Takayuki Iwamoto, Dongwei Zhang, Li Shen, Yuko Takahashi, Arvind Rao, Alastair Thompson, Subrata Sen, Naoto T. Ueno

**Affiliations:** 1 Department of Translational Molecular Pathology, The University of Texas MD Anderson Cancer Center, Houston, Texas USA; 2 Department of Breast Medical Oncology, The University of Texas MD Anderson Cancer Center, Houston, Texas USA; 3 Morgan Welch Inflammatory Breast Cancer Research Program and Clinic, The University of Texas MD Anderson Cancer Center, Houston, Texas USA; 4 Department of Bioinformatics and Computational Biology, The University of Texas MD Anderson Cancer Center, Houston, Texas USA; 5 Department of Breast Surgical Oncology, The University of Texas MD Anderson Cancer Center, Houston, Texas USA; 6 Department of Breast and Endocrine Surgery, Okayama University Hospital, Okayama, Japan

## Abstract

Inflammatory breast cancer (IBC) is a rare subtype of breast cancer, accounting for 8–10% of breast cancer-associated deaths in the US. Clinical hallmarks of IBC include tumor emboli in lymphatic vessels and E-cadherin overexpression, which supports a type of metastasis referred to as cell cluster-based metastasis, prevalent in IBC. In contrast, we previously reported epithelial-to-mesenchymal transition (EMT)-based progression of IBC, utilizing *in vivo* xenografts and *in vitro* Matrigel culture models. To address these two contradictory concepts of IBC metastasis, we used Matrigel culture to induce EMT in a panel of IBC cells. Results revealed Matrigel culture induced vimentin expression in SUM149 and SUM190 IBC cells at the transcriptional and protein levels while maintaining the expression of E-cadherin, a phenomenon referred to as partial EMT. Transcriptional profiling revealed that expression of colony-stimulating factor 1 (CSF-1) was induced in Matrigel culture. When the receptor tyrosine kinase of CSF-1 (CSF-1R) was inhibited by CSF-1R inhibitor BLZ945, the partial EMT was reversed in a dose-dependent manner, indicating that the CSF-1/CSF-1R axis plays a key role in controlling partial EMT. This observation may help reconcile the two contradictory theories of IBC metastasis, EMT vs cell cluster-based metastasis.

## Introduction

Inflammatory breast cancer (IBC) is a rare subtype of breast cancer, accounting for only 2% of all new breast cancer cases, but a clinically dismal disease responsible for 8–10% of all breast cancer-related deaths in the US^1,2^. IBC is diagnosed on the basis of unique clinical presentations, such as skin edema and redness of skin called “peau d’orange,” in addition to pathological findings of invasive cancer^3–5^. There are also other pathological and molecular characteristics unique to IBC that are considered supplemental evidence for its diagnosis. These include intra-lymphatic tumor cell emboli and overexpression of E-cadherin (up to 90% of all IBC cases)^6–8^. Tumor emboli are composed of clustered IBC cells that also express high levels of E-cadherin, a molecule critical for intercellular adhesion. Given this evidence, IBC’s spread has been suggested to occur through collective invasion, a type of invasion in which cancer cells maintain their attachment to each other rather than invading as solitary cells, and then undergo cell cluster-based metastasis by maintaining expression of E-cadherin through the entire process. This concept of metastasis has been suggested in other tumor types as well^9–13^, and has been recapitulated in an IBC xenograft model, with tumor cell emboli and expression of E-cadherin in mouse lymphatic vessels^14^.

The observations on cell cluster-based metastasis contradict the conventionally accepted model of tumor metastasis involving epithelial-to-mesenchymal transition (EMT), during which cancer cells lose expression of E-cadherin, with consequent loss of intercellular adhesions, and gain expression of mesenchymal markers (e.g. vimentin) along with the relevant transcriptional factors (e.g. Twist1 and Zeb1)^15–17^. In contrast to the findings supporting cell cluster-based metastasis in IBC, we previously reported that SUM149 IBC cells underwent EMT in Matrigel culture *in vitro* and metastasized to the lung through the EMT mechanism *in vivo* in a mouse SUM149 xenograft model^18^. Furthermore, *in vivo* metastasis and EMT were inhibited by erlotinib, an inhibitor of epidermal growth factor receptor (EGFR), a molecule known to drive EMT depending on the type of cells, even though the erlotinib dose used in this experiment did not inhibit cell growth. Therefore, it appears that a transient EMT induction plays a role in promoting IBC metastasis, at least in some instances, as reflected in the SUM149 model.

In this scenario, it is important to investigate whether IBC metastasis involves both a cell cluster-based as well as an EMT-mediated process. It has been proposed that IBC primarily undergoes cell cluster-based dissemination but also has plasticity that allows cells to maintain both epithelial and mesenchymal features in a fine-tuned phenotypic balance^19^. Interestingly, emerging evidence implies that cells that have both epithelial and mesenchymal phenotypes, called a “hybrid E/M phenotype,” are more aggressive and metastatic than cells that have either an epithelial or a mesenchymal phenotype^20–22^. However, experimental models to recapitulate the EMT phenotype reflecting dynamic change between epithelial and mesenchymal characteristics are yet to be developed, and the pathological significance of such phenotypes in IBC remains unknown.

We hypothesized that IBC cells, while undergoing invasion in clusters, also transit toward the mesenchymal phenotype at the matrix-enriched tumor periphery at an early stage of metastasis. In the present study, we specifically addressed whether Matrigel culture, simulating the tumor periphery or a microenvironment rich in extracellular matrix, induces a transition toward mesenchymal cells and, if so, whether this phenotypic transition could be inhibited by targeting a key molecular axis involved in the process. The findings suggest that the CSF-1/CSF-1R axis plays an important role in the development of a hybrid E/M phenotype and cellular aggregates associated with metastasis in a subset of IBC. Therapeutic targeting of the CSF-1/CSF-1R axis, therefore, appears to be a rational approach for treatment of these IBC cases and warrants further investigation in clinical settings.

## Results

### Epithelial-phenotype inflammatory breast cancer cells transform to hybrid epithelial/mesenchymal phenotype in Matrigel culture

**Figure 1:**
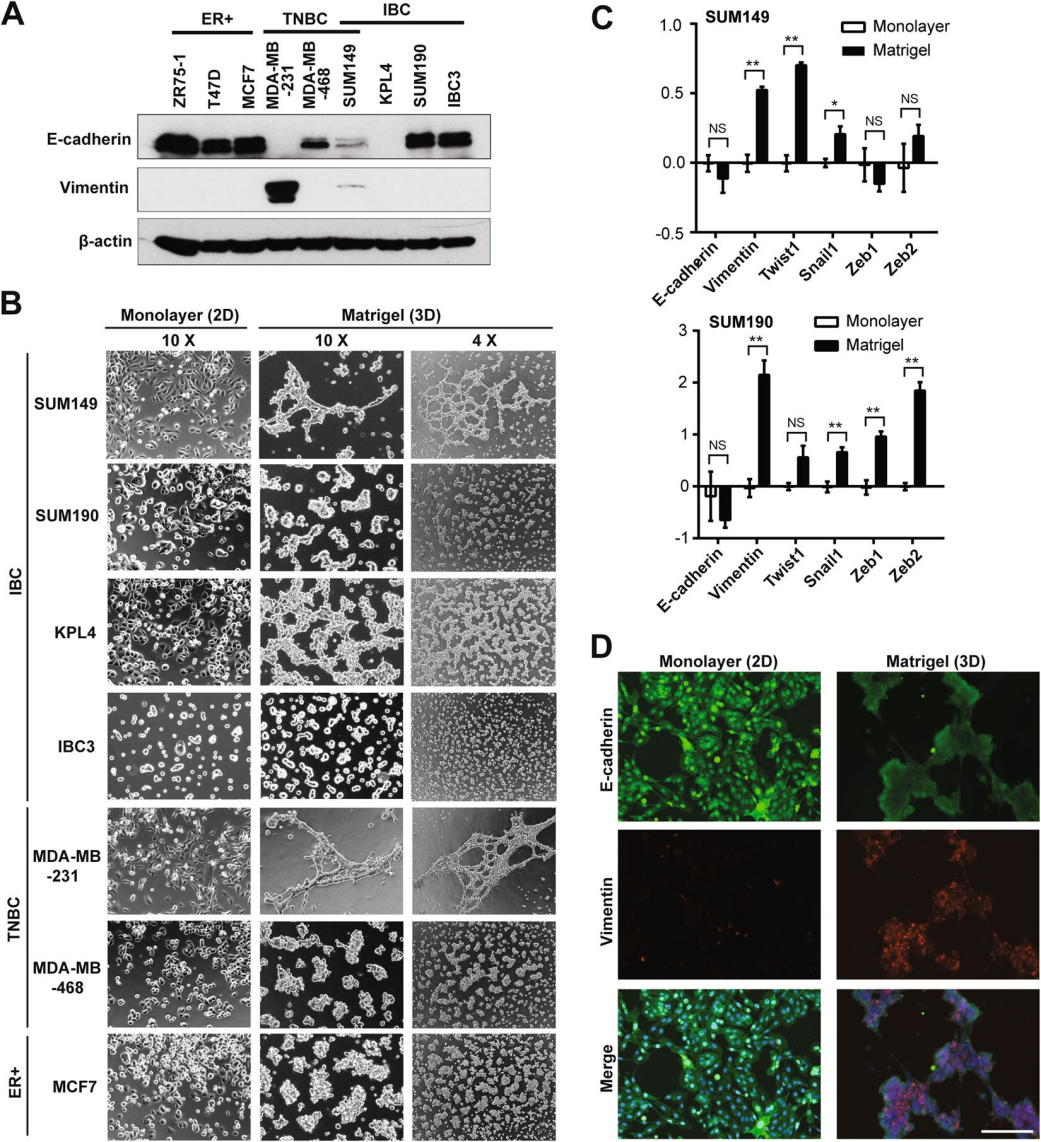
*In vitro* epithelial-to-mesenchymal transition (EMT) induced by Matrigel culture in inflammatory breast cancer. (**A**) Western blots for E-cadherin, vimentin, and β-actin (a loading control) in a panel of breast cancer cells. ER+, estrogen receptor-positive; TNBC, triple-negative breast cancer; IBC, inflammatory breast cancer. SUM149 is an IBC cell line with triple-negative characteristics. Full-length blots with different exposure times are presented in Supplementary Figures S1 and S2. (**B**) Bright-field images depicting typical morphologies of breast cancer cells cultured in monolayer or Matrigel culture conditions. The left 2 columns and the right column are the images captured by objective lens with 10X and 4X magnifications, respectively. (**C**) Transcriptional profiling of EMT markers in IBC cells cultured in monolayer or Matrigel culture conditions, assayed by quantitative RT-PCR. For each marker, the samples from monolayer culture conditions were set as normalizers, and all relative expression values were log2 transformed. Bars, standard error of the mean. (**D)** Immunofluorescent images depicting expression of E-cadherin (green) and vimentin (red) in SUM149 cells cultured in monolayer or Matrigel culture conditions. Merged images are derived from the images of E-cadherin, vimentin, as well as nuclear counterstaining Hoechst 33342 (blue). Bar, 200 µm.

To understand the intrinsic epithelial or mesenchymal characteristics of IBC cells, we first quantified protein levels of epithelial marker E-cadherin and mesenchymal marker vimentin in IBC cell lines as well as in cell lines of other breast cancer subtypes: estrogen receptor-positive (ER+) and triple-negative breast cancer (TNBC) (Fig. 1A, Supplementary Figs S1, S2). As expected, all ER+ breast cancer cells expressed higher levels of E-cadherin with undetectable vimentin. Two TNBC cell lines showed opposite phenotypes; MDA-MB-468 cells expressed E-cadherin but not vimentin, and MDA-MB-231 cells expressed vimentin but not E-cadherin. All IBC cells except for KPL4 cells expressed E-cadherin. SUM149 IBC cells, which are triple-negative, were the only IBC cell line that expressed vimentin at a very low level in addition to E-cadherin, thus representing a hybrid E/M phenotype.

We next characterized morphological phenotypes of IBC cells as well as ER+ and TNBC cells cultured in monolayer and Matrigel cultures (Fig. 1B). In monolayer culture, the TNBC cell line MDA-MB-231 was the only cell line that showed a typical spindle-shaped morphology and scattered spreading pattern (Fig. 1B). In concordance with its low vimentin expression, SUM149 cells contained mixed cell types: epithelial and mesenchymal (or spindle-shaped) in monolayer culture. Other E-cadherin-expressing IBC cell lines, SUM190 and IBC3, showed epithelial-like compacted colonies in monolayer culture. In Matrigel culture, E-cadherin-expressing cell lines except for SUM149 cells showed isolated cell colonies. In contrast to this phenotype, SUM149 cells presented a branching phenotype, which was similar to that of MDA-MB-231 TNBC cells, that were E-cadherin negative. To determine whether this phenotypic conversion observed between SUM149 cells cultured in monolayer and those in Matrigel culture was EMT, we performed transcriptional expression analyses for EMT markers such as E-cadherin and vimentin. We observed the downregulation of E-cadherin and the upregulation of vimentin in not only SUM149 cells but also in the other E-cadherin-expressing IBC cell lines, SUM190 and IBC3, in Matrigel culture compared to monolayer culture (Fig. 1C, Supplementary Fig. S3). We also investigated these EMT marker changes in SUM149 cells at the protein level by immunofluorescence microscopy and observed clear induction of vimentin and slightly reduced E-cadherin expression when grown in Matrigel culture (compared to expression in monolayer culture) (Fig. 1D). The same trend was seen in SUM190 cells although the induction of vimentin was relatively more subtle (Supplementary Fig. S4). Taken together, E-cadherin-expressing (or E phenotype) IBC cells had plasticity to transit into vimentin-expressing (or E/M phenotype) cells under conditions rich in extracellular matrix (i.e., in Matrigel culture).

### Matrigel culture of SUM149 cells induces transcriptional expression changes that represent EMT and inflammatory response

**Figure 2:**
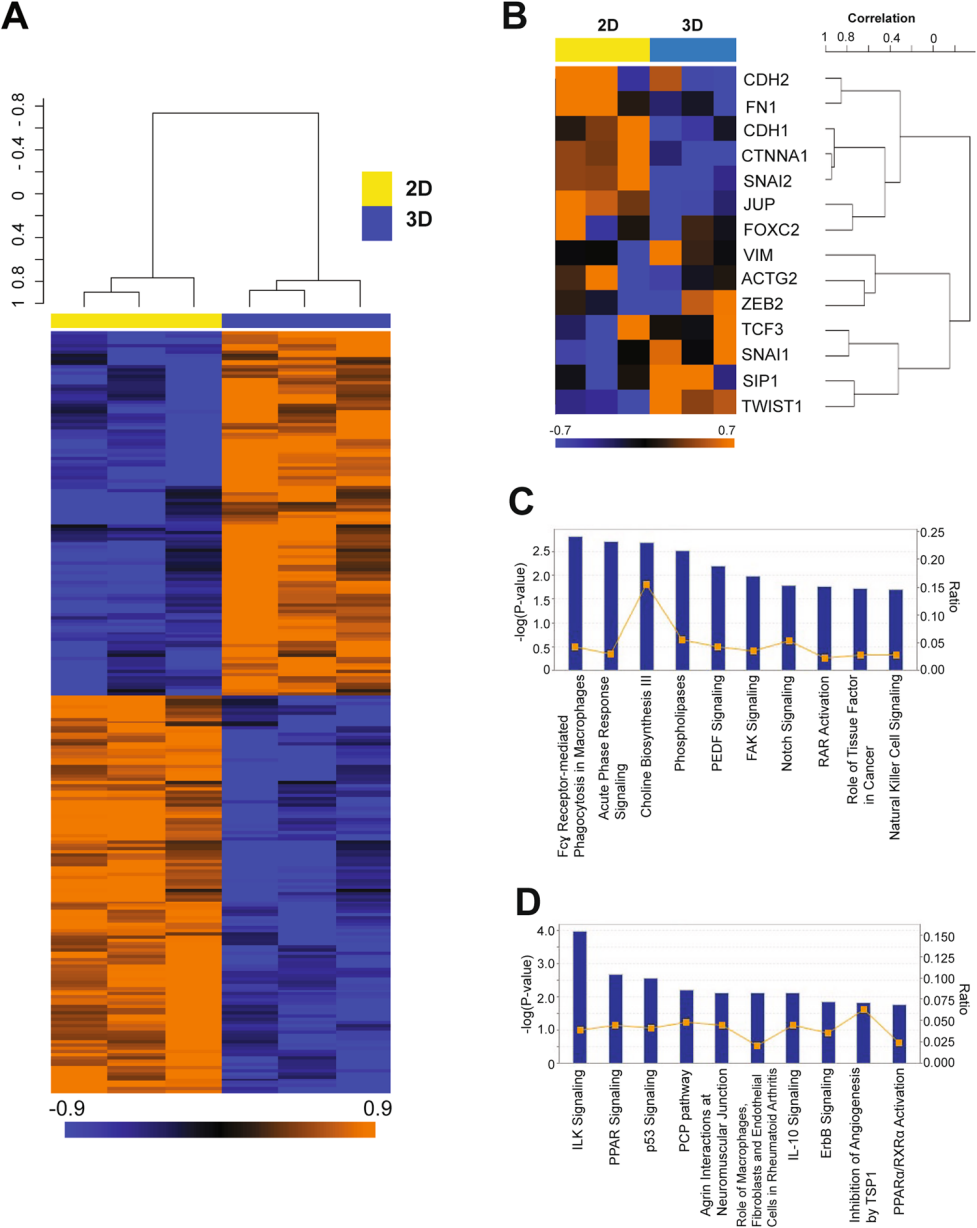
Differentially expressed genes in Matrigel-cultured SUM149 cells represent EMT and inflammatory response pathways. (**A**) Heatmap of transcripts from monolayer- and Matrigel-cultured SUM149 cells (a triplicate sample from each) showing the 152 differentially expressed probe sets between these two conditions. Orange and blue indicate higher and lower relative expression, respectively. (**B**) Heatmap of EMT-related transcripts from monolayer- and Matrigel-cultured SUM149 cells (a triplicate sample from each). (**C**,**D**) Canonical pathway analyses by Ingenuity pathway analysis (IPA) software of overexpressed (**C**) and underexpressed (**D**) genes in Matrigel-cultured SUM149 cells compared to monolayer-cultured SUM149 cells. Annotated pathways were ranked according to the values of −log (*P*-value).

We next performed gene expression microarray analysis of SUM149 cells cultured in monolayer and Matrigel cultures. The objectives of this study were twofold: 1) confirming induction of the EMT gene signature in Matrigel-cultured SUM149 cells at the whole-genome level, and 2) identifying novel targets to inhibit EMT in E-cadherin-expressing IBC cells. For this analysis, we selected 242 probe sets (corresponding to 242 unique genes) that showed statistically significant differential expression patterns between the two culture conditions (with a *p* value of <0.01 and a fold change of >1.5). We defined these genes as the Matrigel gene signature. A heatmap using this gene signature is shown in Fig. 2A, and all genes in the Matrigel gene signature are listed in Supplementary Table S1. Genes upregulated in Matrigel culture included colony stimulating factor 1 (*CSF-1*) and angiopoietin 1 (*ANGPT1*), both of which have important roles in inflammatory response and metastasis^23,24^. In contrast, the genes that represent epithelial features, such as keratin 18 (*KRT18*) and claudin 1 (*CLDN1*)^25^, were downregulated in Matrigel culture (Supplementary Table S1).

We next performed gene set enrichment analysis (GSEA) to elucidate the biological significance of upregulated and downregulated genes in Matrigel culture compared with monolayer culture^26,27^. The Gene Ontology gene sets “regulation of cell-cell adhesion” and “positive regulation of cell-cell adhesion” were downregulated in Matrigel-cultured SUM149 cells (Supplementary Table S2), suggesting that SUM149 cells were losing cell-cell adhesion in Matrigel culture. In contrast, gene sets associated with metabolic processes (e.g., oxidoreductase activity, cholesterol metabolic process, and acetyl-CoA metabolic process) and inflammatory response processes (e.g., cytokine receptor activity, chemokine receptor activity, arachidonic acid metabolic process) were upregulated in Matrigel-cultured SUM149 cells (Supplementary Table S2). These findings suggest that Matrigel culture mimics the perturbed metabolic status and inflammatory milieu of the IBC tumor microenvironment. We also used specific gene sets (Supplementary Table S3) that represent aggressive features of breast cancer, having been reported to correlate with TNBC, for another series of GSEA. According to previous reports, these gene sets are annotated as follows: EMT^28–30^, claudin-low (CL)^31^, immune kinase (IKS)^32^, mitosis kinase (MKS)^32^, and genomic grade index (GGI)^33^ (Supplementary Table S3). We identified a significant correlation with the EMT gene set but not with the others, including the proliferation-related gene set (i.e., GGI) (Table 1). Among the EMT gene set, we confirmed that Twist1 and Snail1, both of which are EMT-driving transcription factors^16,17^, were upregulated and that E-cadherin (CDH1) and plakoglobin (JUP), which are components of adhesion junctions and desmosomes^34^, were downregulated in Matrigel culture (Fig. 2B). These findings support the hypothesis that E-cadherin-expressing IBC cells undergo EMT in Matrigel culture while E-cadherin protein is still expressed.

**Table 1:** Gene set enrichment analysis of differentially expressed genes in SUM149 cells cultured in Matrigel compared to monolayer using gene sets clinically relevant to TNBC.

Gene set	Number of genes	Efron-Tibshirani’s GSA test *p* value	Direction of overexpression
EMT	14	<0.001	Matrigel
IKS	15	0.466	Matrigel
MKS	12	0.397	Matrigel
CL	19	0.096	Monolayer
GGI	128	0.21	Monolayer

### Matrigel culture induces the innate immune response pathway and downregulates the p53 pathway in SUM149 cells

We next performed Ingenuity pathway analysis (IPA) to further understand the molecular background of Matrigel culture–induced EMT in SUM149 cells. Genes that were upregulated and downregulated in Matrigel-cultured SUM149 cells compared to those from monolayer culture were incorporated into IPA independently, and canonical pathway analysis was performed as previously described^35^. The top-ranked pathway for upregulated genes was Fcɣ receptor–mediated phagocytosis in macrophages (Fig. 2C). Upregulated genes were also correlated with an innate immune response-related pathway, natural killer cell signaling. In contrast, downregulated genes were represented by p53 signaling (Fig. 2D), suggesting that the p53 pathway is downregulated in Matrigel-cultured SUM149 cells. These findings suggest that Matrigel culture induced innate immune responses in SUM149 cells, as observed in macrophages and natural killer cells, concurrently downregulated the p53 pathway.

### Overexpressed genes in the Matrigel gene signature reflect inflammatory response in TNBC

Breast cancer is a heterogeneous disease in terms of EMT-associated markers reflecting prognosis of each subtype^36–38^. TNBC is known to have more mesenchymal (or EMT-like) characteristics coupled with poor prognosis in general when compared to ER+ and HER2+ breast cancers^36–38^. Recently, using an RNA *in situ* hybridization (ISH) assay that targets both epithelial and mesenchymal markers, Yu *et al*. surprisingly showed that the EMT characteristics being reported in TNBC can be attributed to a higher percentage of E/M hybrid breast cancer cells in TNBC compared to the other subtypes, ER/PgR+ and HER2+ , in which epithelial phenotype cells were dominant^21^. Another study also showed that the E/M hybrid gene signature correlated with poor survival outcome in breast cancer patients^22^. Based on these studies, we hypothesized that E/M phenotype-driving genes could be enriched in TNBC and such genes could be overlapping with the Matrigel gene signature because of the overlapping nature of an E/M hybrid phenotype.

**Figure 3:**
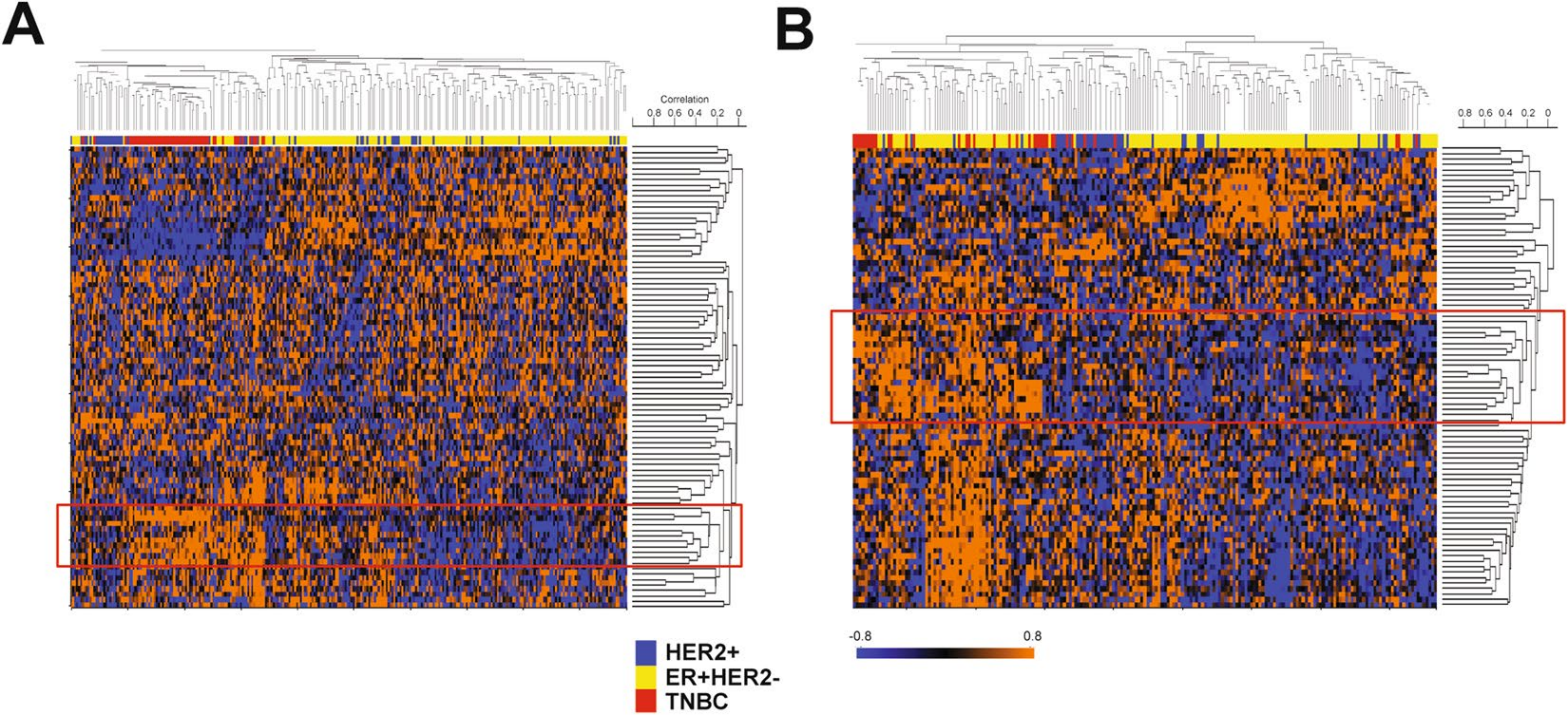
Gene signature derived from Matrigel-cultured SUM149 cells represents activated inflammatory response genes in human TNBC. (**A**,**B**) Heatmaps of Wang (A) and MDA233 (B) breast cancer data sets with overexpressed genes in Matrigel-cultured SUM149 cells. Unsupervised hierarchical clustering was applied to draw these heatmaps. Before the clustering, patients in each data set were categorized into 3 subgroups: ER+ HER2−, HER2+, and TNBC, according to the transcriptional levels of ESR1 and ERBB2 (or HER2) as previously described^40^. Red boxes denote gene clusters that were highly correlated with TNBC sample clusters. Orange and blue indicate higher and lower relative expression, respectively.

To identify such genes, we clustered two independent breast cancer data sets that have gene expression data and subtype information using the genes overexpressed in Matrigel-cultured SUM149 cells (or overexpressed genes from the Matrigel gene signature). The breast cancer data sets used were the Wang and MDA233 data sets^39,40^. The subtypes (ER+ HER−, HER2+, TNBC) were reasonably distributed in the expected proportions in both data sets (data not shown). With the Wang data set, which contains survival information, we confirmed differences in terms of recurrence-free survival among the subtypes when 48 months after surgery was set as the endpoint of follow-up for all cases (*p* = 0.038, log rank test) (Supplementary Fig. S5). Therefore, these two data sets are representative cohorts of breast cancers that have molecular and clinical heterogeneity. We adopted unsupervised rather than supervised clustering because in the Yu *et al*. study, the E/M hybrid phenotype was not necessarily exclusive to TNBC but also seen in ER/PgE+ and HER2+ breast cancer, although E/M hybrid phenotype cells were relatively rare in the latter subtypes^21^. With these clusterings, we found independent gene clusters overexpressed in a significant proportion of TNBCs in both the Wang and MDA233 data sets (Fig. 3A,B).

Although these genes are overexpressed in a subset of other subtypes as well, given the concept being developed above, we selected these two independent gene clusters as candidate genes for further study. We consequently found that 20 genes overlapped between these two independent gene clusters (Table 2), and the majority of them were directly or indirectly correlated with inflammatory response pathways. Colony stimulating factor 1 (CSF-1) and complement C3a receptor 1 (C3AR1) were among these 20 genes^41–43^. When expression of these 20 genes was compared by *t*-test between TNBC and non-TNBC (i.e. ER+ HER2−, HER2+, TN), most genes were overexpressed in TNBC with statistical significance (*p* < 0.05) in either or both of the two breast cancer data sets (Supplementary Table S4).

**Table 2:** Matrigel culture genes overexpressed in human TNBC.

Probe	Gene symbol	Gene name
220122_at	*MCTP1*	Multiple C2 domains, transmembrane 1
205348_s_at	*DYNC1I1*	Dynein, cytoplasmic 1, intermediate chain 1
210605_s_at	*MFGE8*	Milk fat globule-EGF factor 8 protein
220559_at	*EN1*	Engrailed homeobox 1
209716_at	*CSF1*	Colony stimulating factor 1 (macrophage)
204457_s_at	*GAS1*	Growth arrest-specific 1
210145_at	*PLA2G4A*	Phospholipase A2, group IVA (cytosolic, calcium-dependent)
209821_at	*IL33*	Interleukin 33
215223_s_at	*SOD2*	Superoxide dismutase 2, mitochondrial
204122_at	*TYROBP*	TYRO protein tyrosine kinase binding protein
209906_at	*C3AR1*	Complement component 3a receptor 1
206060_s_at	*PTPN22*	Protein tyrosine phosphatase, non-receptor type 22 (lymphoid)
218723_s_at	*RGCC*	Regulator of cell cycle
209584_x_at	*APOBEC3C*	Apolipoprotein B mRNA editing enzyme, catalytic polypeptide-like 3C
204646_at	*DPYD*	Dihydropyrimidine dehydrogenase
210786_s_at	*FLI1*	Friend leukemia virus integration 1
219563_at	*C14orf139*	Chromosome 14 open reading frame 139
219387_at	*CCDC88A*	Coiled-coil domain containing 88A
210073_at	*ST8SIA1*	ST8 alpha-N-acetyl-neuraminide alpha-2,8-sialyltransferase 1
218319_at	*PELI1*	Pellino homolog 1 (*Drosophila*)

### Targeting the CSF-1/CSF-1R axis inhibits EMT of SUM149 cells cultured in Matrigel

**Figure 4:**
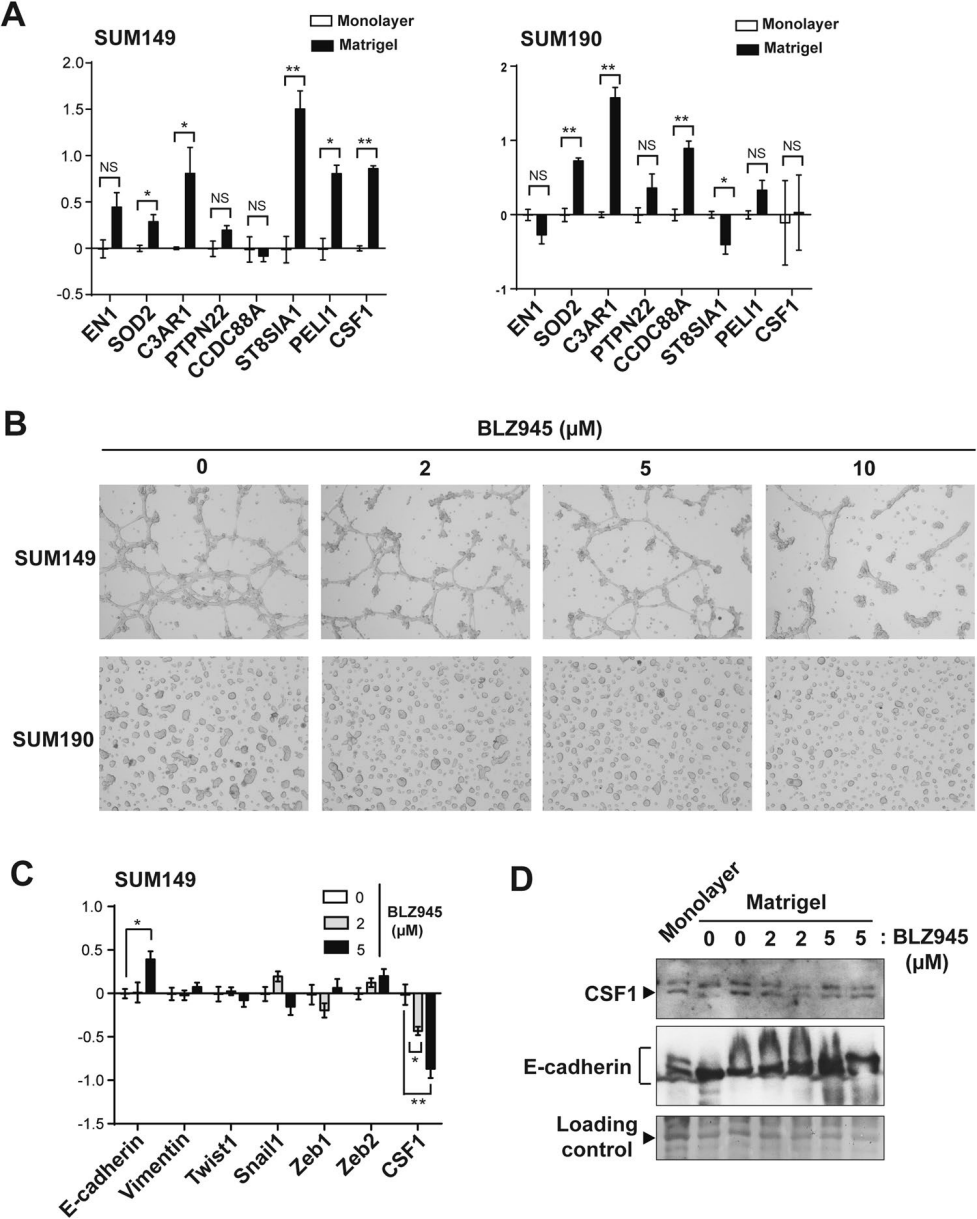
Colony stimulating factor 1 (CSF-1) is associated with *in vitro* EMT in IBC cells. (**A**) Transcriptional profiling of inflammation-related genes in SUM149 and SUM190 IBC cells cultured in monolayer and Matrigel culture conditions, assayed by quantitative RT-PCR. For each marker, the samples from monolayer culture conditions were set as normalizers, and all relative expression values were log2 transformed. Bars, standard error of the mean. (**B**) Bright-field images depicting typical morphologies of SUM149 and SUM190 cells cultured in Matrigel culture conditions treated with different concentrations of a CSF-1 receptor inhibitor, BLZ945. (**C**) Transcriptional profiling of EMT markers in Matrigel-cultured SUM149 cells treated with different concentrations of BLZ945, assayed by quantitative RT-PCR. For each marker, the samples from untreated controls were set as normalizers, and all relative expression values were log2 transformed. Bars, standard error of the mean. (**D**) Western blots for CSF-1 and E-cadherin in SUM149 cells cultured in monolayer or Matrigel, in which SUM149 cells were treated with BLZ945 (0, 2, or 5 µM in the final concentration). The bands (at around 48 kDa) stained with Ponceau S are shown as a loading control. Full-length blots with different exposure times are presented in Suplementary Fig. S11.

Given these inflammatory genes, including CSF-1 and C3AR1, were identified based on the concept of an E/M hybrid phenotype, we hypothesized that these inflammatory-related genes could be drivers of the E/M phenotype in the IBC cells cultured in Matrigel. To test this hypothesis, we first selected 8 inflammation-related genes and ran quantitative RT-PCR analysis to validate the overexpression of these genes in Matrigel-cultured SUM149 and SUM190 cells compared to those in monolayer culture (Fig. 4A). We confirmed a varying degree of overexpression of 7 and 6 genes in Matrigel-cultured SUM149 and SUM190 cells, respectively, compared to their monolayer-cultured counterparts. In contrast, we observed overexpression of only 2 genes in KPL4 cells (CCDC88A and ST8SIA1) and 1 gene in IBC3 cells (SOD2) (Supplementary Fig. S6); CSF-1 and C3AR1 were not overexpressed in these cell lines. This finding suggests that different IBC cell lines behave differently in Matrigel culture and that CSF-1 or C3AR1 may play a key role in only a subset of IBCs.

Since CSF-1 and C3AR1 are directly involved in inflammatory response pathways and there are commercially available inhibitors targeting pathways involving these molecules^43,44^, we tested inhibitors against CSF-1R, a receptor for CSF-1, and C3AR1 in Matrigel-cultured SUM149 and SUM190 cells to see whether these drugs inhibit EMT in IBC cells in Matrigel culture. C3AR1 inhibitor SB290157 did not inhibit the EMT of SUM149 cells cultured in Matrigel (data not shown)^43^. We therefore focused on the CSF-1/CSF-1R axis and next studied the expression of CSF-1 at the transcriptional and protein levels in a panel of breast cancer cells. CSF-1 was expressed at varying transcriptional and protein levels across all types of breast cancers (Supplementary Figs S7, S8, S9). However, TNBC cell lines MDA-MB-231 and MDA-MB-468 and IBC cell lines KPL4 and IBC3 relatively overexpressed CSF-1 compared to SUM149 although SUM149 also expressed CSF-1 protein at a detectable level (Supplementary Fig. S7). Together with a previous study that showed varying levels of CSF-1R expression across all types of breast cancer^45^, these findings suggest that the CSF-1/CSF-1R axis has a functional role in breast cancer, including IBCs.

Next, to test the hypothesis that the CSF-1/CSF-1R axis is a driver of the E/M hybrid phenotype in the IBC cells cultured in Matrigel, we treated Matrigel-cultured SUM149 IBC cells with CSF-1R inhibitor BLZ945. We observed inhibition of branching and spindle cell-like phenotypes in Matrigel-cultured SUM149 cells with increasing dose of BLZ945 (Fig. 4B)^44,46^. We also observed much smaller and scattered colonies in BLZ945-treated Matrigel-cultured SUM190 cells with increasing dose of BLZ945. Cellular analyses at a single-cell level revealed that BLZ945 significantly changed the morphology of cells starting at the lowest concentration of 2 µM up to 10 µM (Supplementary Fig. S10). Molecularly, we also observed partial reversion of EMT with BLZ945 treatment in Matrigel-cultured SUM149 cells (Fig. 4C), while the effect was less pronounced with SUM190 cells (data not shown). Upregulation of E-cadherin was evident with 5 µM of BLZ945 in Matrigel-cultured SUM149 cells compared to untreated control cells at both the transcriptional and protein levels (Fig. 4C,D, and Supplementary Fig. S11). In addition, Twist1 and Snail1 were downregulated with 5 µM of BLZ945 at the transcriptional level although these changes were not statistically significant. These data suggest that CSF-1R inhibition partially reversed the EMT associated with the expression of CSF-1/CSF-1R axis in Matrigel-cultured SUM149 cells. Interestingly, CSF-1 transcriptional expression was downregulated with BLZ945 in a dose-dependent manner, although the change in protein level was marginal. These data suggest that CSF-1 is a transcriptional target of the CSF-1/CSF-1R pathway and that there could be a positive signaling loop in the pathway that drives the E/M hybrid phenotype in IBC cells.

## Discussion

Using E-cadherin-expressing IBC cells (SUM149 and SUM190) cultured in Matrigel, we studied partial EMT, the transition from an epithelial (E) to hybrid epithelial/mesenchymal (E/M) phenotype, with the goal of obtaining evidence to reconcile two contradictory metastasis theories, cell cluster-based metastasis^9–11^ and EMT-mediated metastasis^15–17^, for IBC cells. We found that SUM149 and SUM190 IBC cells transitioned from an E to E/M phenotype with the induction of CSF-1 expression in Matrigel culture. Following treatment with BLZ945, an inhibitor of the CSF-1R, the partial EMT was reversed in a dose-dependent manner. Thus, we propose that the CSF-1/CSF-1R axis presents an actionable target for inhibition of partial EMT (hence metastasis) in some IBCs.

Based on theoretical and experimental studies, Jolly *et al*. proposed that there is a phenotypic state in IBC cells that represents both epithelial and mesenchymal properties, referred to as the hybrid E/M phenotype^19,20,47^. The hybrid E/M phenotype has been reported to be an aggressive and metastatic feature of tumors^20,21^. In Matrigel culture, we observed clear induction of vimentin with varying degrees of E-cadherin expression in SUM149 and SUM190 cells (Fig. 1C,D), indicating that this culture condition induces lineage transition toward the hybrid E/M phenotype, or partial EMT. This molecular transition observed in Matrigel (compared to monolayer culture) was accompanied by morphological transition represented by branching and spindle cell-like phenotypes, previously defined by us as an *in vitro* EMT phenotype^18^. Kenny *et al*. previously screened morphological characteristics of Matrigel-cultured breast cancer cells and found 4 distinct phenotypes^48^: round, mass, grape-like, and stellate. According to this grouping, SUM149 cells are categorized into the stellate type, which corresponds to our “branching and spindle cell-like” phenotype. In their study, 4 cell lines, all of the TNBC subtype, were categorized into the stellate phenotype: BT-549, Hs578T, MDA-MB-231, and MDA-MB-436^48^. However, none of these 4 cell types expressed E-cadherin, even in monolayer culture. Therefore, to the best of our knowledge, our studies present the first report of morphological transition from an epithelial to mesenchymal-like phenotype in Matrigel culture^18^. This model is novel in enabling induction of a hybrid E/M phenotype, a putative metastatic cell population, from a bulk IBC cell population that expresses E-cadherin but not vimentin. This model could offer a unique opportunity to the IBC research community to molecularly dissect the pathways associated with the development of the hybrid E/M phenotype associated with IBC metastasis^19^.

EMT is a transcription factor–driven cellular program, verified by downregulation of E-cadherin, expression of EMT-driving transcription factors, cytoskeletal reorganization (e.g., expression of vimentin), and secretion of fibronectin^15–17,49^. In the present model, E-cadherin and vimentin were transcriptionally downregulated and upregulated, respectively. Moreover, some EMT-driving transcription factors (e.g., Twist1 and Snail1) were overexpressed in Matrigel-cultured SUM149 and SUM190 cells (Figs 1C and 2B). Since experimental overexpression of individual EMT-driving transcription factors, such as Twist1 and Foxc2, has been reported as sufficient to drive EMT in mammary cells^30,50,51^, it is likely that Matrigel culture induced EMT-like molecular changes in SUM149 and SUM190 cells. However, we also observed a variance in terms of expected changes in EMT markers. For instance, at the transcriptional level, the induction of fibronectin was not observed in Matrigel culture (Fig. 2B). This may be explained by the fact that Matrigel is highly enriched with extracellular matrix proteins, including laminin and collagen IV, both of which can bind to integrin receptor family proteins and activate intracellular signaling, which could compensate for the role of fibronectin^52–54^. Our finding also reinforces reported evidence that EMT induction is not always complete but that there can be intermediate or partial EMT^19,20,47^. EMT is also physiologically induced by extrinsic soluble factors, including TGF-β and TNF-α^55,56^. Since we could not induce EMT in SUM149 cells with TGF-β and/or TNF-α in monolayer culture (data not shown), the extracellular matrix may be indispensable for inducing EMT in SUM149 IBC cells. These findings together support that EMT in IBC cells, at least to the varying degrees detected in SUM149 and SUM190 cells, depends on the extracellular matrix (or Matrigel *in vitro*) and is driven toward a hybrid E/M phenotype rather than to an exclusively mesenchymal phenotype.

Among overrepresented pathways observed in Matrigel-cultured SUM149 cells (with IPA analysis, Fig. 2C), Fcɣ receptor–mediated phagocytosis in macrophages and natural killer cell signaling are relevant to immune responses to pathogenic viruses or bacteria^57,58^. Biliary epithelial cells are known to transform to mesenchymal cells by utilizing such immune responses to infective agents such as double-stranded RNA viruses^59^. Therefore, analogous to this phenomenon, we assume that SUM149 cells utilize immune response pathways while undergoing partial EMT. In addition to immune response pathways, the downregulated p53 pathway in Matrigel culture may also help in promoting the EMT process (Fig. 2D), as reported in several models^60–62^.

CSF-1 is a ligand for the receptor tyrosine kinase CSF-1R^63^. CSF-1 induces the differentiation commitment of myeloid progenitor cells to non-classic M2 macrophages, which in general play a tumor suppressive role by inhibiting T cell functions in the tumor microenvironment^63,64^. In fact, a study in the late 1980s reported that a CSF-1 infusion into melanoma-bearing mice prevented distant metastasis of melanoma^65^. However, emerging evidence instead supports an oncogenic function of CSF-1^63^. Kubota *et al*. reported that the CSF-1/CSF-1R axis plays a role not only in recruiting macrophages to the tumor microenvironment but also in promoting lymphangiogenesis of tumors, thus promoting tumor growth^66^. Another series of studies also showed that CSF-1 played a role in a biological circuit between stroma cells (e.g. macrophages, mesenchymal stem cells) and tumor cells by promoting invasion and metastasis of tumors^67,68^. Morandi *et al*. demonstrated an autocrine loop of the CSF-1/CSF-1R axis, which promoted proliferation of breast cancer cells through activating the MEK/ERK pathway^45^. We also observed this possible autocrine loop of the CSF-1/CSF-1R axis at the transcriptional level in SUM149 cells (Fig. 4A,C). These findings together suggest that the CSF-1/CSF-1R axis could be a promising therapeutic target for metastasis-prone IBC. Because our Matrigel culture RT-PCR results differed for different IBC cell lines, further investigation of CSF-1 may reveal that certain subtypes of IBC, rather than all IBCs, are aligned with this theory.

**Figure 5:**
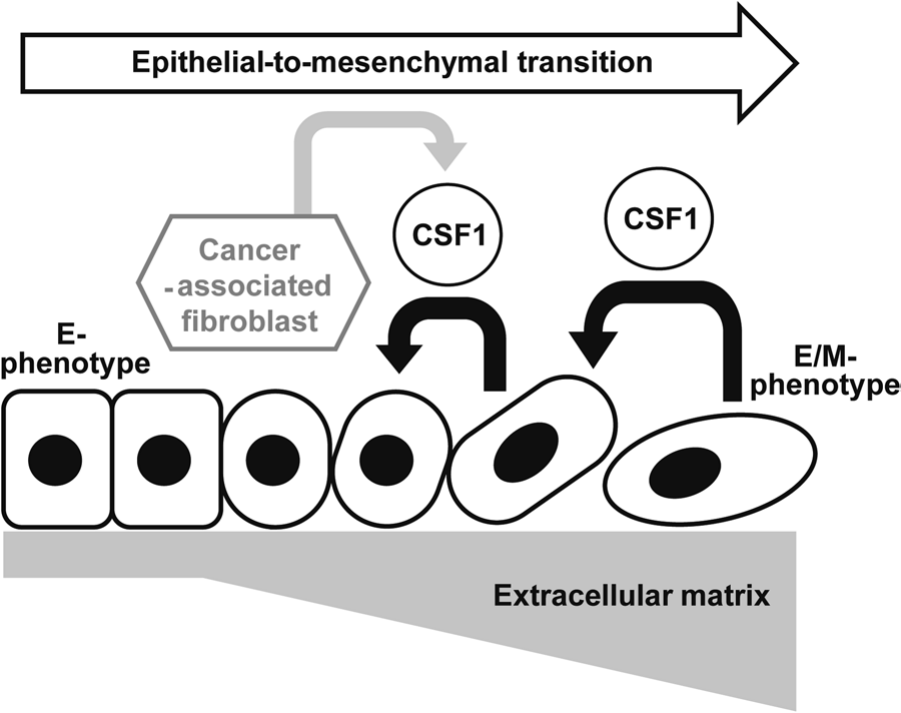
A plausible model of CSF-1-driven EMT from the E to E/M phenotype depending on the extracellular matrix in IBC.

In Matrigel culture, we partially reversed the branching and spindle cell-like phenotype of IBC cells with CSF-1R inhibitor BLZ945 (Fig. 4B). In our previous report, a phenotypic reversal was seen with the metastasis-inhibitory effect *in vivo* observed with EGFR inhibitor erlotinib^18^. Therefore, the present data suggest that CSF-1/CSF-1R axis inhibition is also likely to inhibit IBC metastasis *in vivo* as indicated in other tumor types^45,46,69^. Since CSF-1 is secreted from tumor microenvironment cells, not only mesenchymal stem cells^67^ but also tumor-associated stroma cells and macrophages^63^, an *in vivo* animal model that simulates this complexity is required. Experimental findings from animal models with the endpoints of metastasis inhibition as well as hybrid E/M (or epithelial) phenotype is expected to reveal a specific role of the CSF-1/CSF-1R axis in the IBC microenvironment and metastasis (Fig. 5).

In conclusion, we have established a model in which IBC cells undergo transition from an epithelial to a hybrid E/M phenotype using *in vitro* Matrigel culture and propose that the CSF-1/CSF-1R axis is a promising pathway to be targeted for inhibiting IBC metastasis. This model eventually offers an opportunity to reconcile the relative contributions of two contradictory metastasis theories for IBC, cell cluster and EMT-mediated metastasis.

## Methods

### Cell culture

A panel of 9 human breast cancer cell lines representing ER+, TNBC, and IBC subtypes was used. SUM149 and SUM190 cells were obtained from Asterand Bioscience (Detroit, MI). IBC3 cells were kindly provided by Dr. W. A. Woodward (The University of Texas MD Anderson Cancer Center, Houston, TX)^70^. SUM149, SUM190, and IBC3 cells were maintained in DMEM/Nutrient Mixture F-12 (F12) with 5% FBS, 1X Antibiotic-Antimycotic (AA), 1 mg/mL hydrocortisone, and 5 mg/mL insulin. KPL4 cells were kindly provided by J. Kurebayashi (Kawasaki Medical School, Kurashiki, Japan) as previously described^18^. ZR75-1, T47D, MCF7, MDA-MB-231, and MDA-MB-468 cells were all obtained from American Type Culture Collection (Manassas, VA)^71^. KPL4, ZR75-1, T47D, MCF7, MDA-MB-231, and MDA-MB-468 cells were all maintained with DMEM/F12 supplemented with 10% FBS, 1X AA. SUM149, SUM190, MDA-MB-231, and MDA-MB-468 cells were authenticated in 2014. MCF7 cells were authenticated in 2010. ZR75-1, T47D, and KPL4 cells have not been authenticated yet. Cell line authentication was performed using short tandem repeat methodology conducted by the Characterized Cell Line Core Facility at The University of Texas MD Anderson Cancer Center as previously described^71^.

### siRNA transfection

MDA-MB-231 cells were transfected with a Silencer Select Pre-Designed siRNA to CSF-1 (Thermo Fisher Scientific, Waltham, MA) at the final concentrations of 2, 10, 25, and 50 nmol/L with use of Lipofectamine RNAi Max reagent (Invitrogen, Carlsbad, CA).

### Western blot analysis

Cells were lysed with radioimmunoprecipitation assay (RIPA) buffer, resolved by sodium dodecyl sulfate–polyacrylamide gel electrophoresis, and transferred to nitrocellulose membranes (Bio-Rad Laboratories, Hercules, CA), and the membranes were incubated with primary antibodies as previously described^71,72^. Primary antibodies used for western blots were as follows: anti-E-cadherin mouse monoclonal antibody (Ab) (clone 36/E-cadherin) from BD Biosciences (San Jose, CA), anti-vimentin rabbit monoclonal Ab (clone D21H3) and anti-β-actin rabbit monoclonal Ab (clone 13E5) from Cell Signaling Technology (Danvers, MA), and anti-CSF-1 rabbit polyclonal Ab from Abcam (Cambridge, UK). Primary antibodies were diluted at the ratio of 1:1,000. The secondary antibodies, anti-rabbit and anti-mouse IgG-horseradish peroxidase (HRP) conjugated, were purchased from GenDEPOT (Barker, TX) and diluted at the ratio of 1:2,000. Signals were developed with ECL western blotting detection reagent (GE Healthcare, Little Chalfont, UK) and detected using autoradiography films (Denville Scientific Inc., Metuchen, NJ).

### Matrigel culture

Matrigel (Corning, Tewksbury, MA) was stored at −80 °C. Before use, Matrigel was thawed in the 4 °C fridge overnight. For the Matrigel coating, 100 µL and 300 µL of Matrigel were applied to each well of 24- and 6-well plates, respectively, and incubated in a 37 °C incubator for 30 min to solidify the Matrigel. Then, 0.5–2.0 × 10^5^ and 0.5–2.0 × 10^6^ cells were resuspended in 0.5 mL and 2.0 mL of culture medium with 2% Matrigel on ice and plated to the solidified bottom layer of the 24- and 6-well plates, respectively. In order to retrieve the cells cultured in Matrigel for the gene expression assay, Cell Recovery Solution (Corning) was used according to the manufacturer’s protocol.

### Morphological and immunofluorescent analysis

To capture the morphological features of breast cancer cells with bright-field images, 24-well tissue culture plates (Corning) were used and images were taken by Olympus IX81 inverted fluorescence microscopy (Olympus, Center Valley, PA). To capture the immunofluorescent images, 24-well glass bottom plates (Greiner Bio-One, Monroe, NC) were used and images were taken by the imaging reader Cytation 3 (BioTek, Winooski, VT). The immunofluorescent and bright field images were analyzed with the data collection and analysis software Gen5 3.02 (BioTek). Anti-E-cadherin mouse monoclonal antibody (clone HECD-1) from Life Technologies (Carlsbad, CA) and anti-vimentin rabbit monoclonal antibody (clone D21H3) from Cell Signaling Technology were used for the immunofluorescent analysis. Alexa Fluor 488 goat anti-mouse IgG and Alexa Fluor 594 goat anti-rabbit IgG secondary antibodies were purchased from Invitrogen (Carlsbad, CA). A standard staining protocol as described previously was used for both Matrigel and monolayer cultured cells^71^. Briefly, cells were fixed with 10% neutralized formalin, and cell membranes were permeabilized with 0.2% Triton X-100. Cells were then incubated with 3% bovine serum albumin in phosphate-buffered saline (PBS) for an hour for the blocking, and incubated overnight with primary antibodies diluted with PBS at the ratio of 1:250. Cells were then incubated for an hour with secondary antibodies diluted with PBS at the ratio of 1:250. Finally, cells were incubated for 5 minutes with Hoechst 33342 dissolved in PBS for nuclear counterstaining. Cells were then immersed in PBS and subjected to immunofluorescent microscopic analysis by Cytation 3.

### mRNA extraction and quantitative RT-PCR analysis

Total RNA was extracted using the RNeasy kit (Qiagen, Hilden, Germany). RNAs were reverse transcribed to complementary DNA using the SuperScript III First-Strand Synthesis System according to the manufacturer’s instructions. cDNAs of the genes of interest were amplified and quantified by SYBR Green-based quantitative PCR using a ViiA7 Real-Time PCR System (Life Technologies). SYBR Green PCR Master Mix was purchased from Life Technologies. Primer pairs were purchased from Sigma-Aldrich (St. Louis, MO), and the sequences of the primer DNA oligos were as follows: E-cadherin-Forward (F), 5′TGAAGATTGCACCGGTCGAC3′; E-cadherin-Reverse (R), GGATGACACAGCGTGAGAGA; Vimentin-F, TTCCAAACTTTTCCTCCCTGAACC; Vimentin-R, TCAAGGTCATCGTGATGCTGAG; Twist1-F, GGACAAGCTGAGCAAGATTCAGA; Twist1-R, GTGAGCCACATAGCTGCAG; Snail1-F, TGCAGGACTCTAATCCAGAGTTT; Snail1-R, GGACAGAGTCCCAGATGAGC; Zeb1-F, GGTCTGATGAAGGATGACAGGGC; Zeb1-R, CTTCAGACACTTGCTCACTACTC; Zeb2-F, GTGACAAGACATTCCAGAAAAGCAG; Zeb2-R, GAGTGAAGCCTTGAGTGCTC; EN1-F, 5′TGGGTGTACTGCACACGTTATTC3′; EN1-R, TGGAACTCCGCCTTGAGTCT; SOD2-F, AAGGGAGATGTTACAGCCCAGATA; SOD2-R, TCCAGAAAATGCTATGATTGATATGAC; C3AR1-F, CCCTACGGCAGGTTCCTATG; C3AR1-R, GACAGCGATCCAGGCTAATGG; PTPN22-F, AGGCAGACAAAACCTATCCTACA; PTPN22-R, TGGGTGGCAATATAAGCCTTG; CCDC88A-F, ATGCCTCACTTAGAATGCACAA; CCDC88A-R, AGACATTTGGCAACGACATCA; ST8SIA1-F, CATGCGATGCAATCTCCCTC; ST8SIA1-R, CTGGGATTAGCTGTCACTAACTG; PELI1-F, AACAAAGACCAGCATAGCAT; PELI1-R, GGTGTTGCTGTCATGAGTAT; CSF1-F, CCTCCCACGACATGGCT; CSF1-R, GAGACTGCAGGTGTCCACTC; GAPDH-F, CTCCTGTTCGACAGTCAGCC; GAPDH-R, ACCAAATCCGTTGACTCCGAC; β-actin-F, CTTCGCGGGCGACGATGC; β-actin-R, CGTACATGGCTGGGGTGTTG. Paired samples (monolayer vs Matrigel, BLZ945 non-treated vs treated) derived from the same cell line were always run on the same plate. For each sample, expression of the gene of interest was adjusted by the expression of an endogenous control gene, either GAPDH or β-actin. In order to compare gene expression levels among paired samples, the gene expression of monolayer-cultured and BLZ945-nontreated samples was used for normalization for each gene of interest, and the gene expression of Matrigel-cultured and BLZ945-treated samples was presented in terms of fold change in relation to the normalizer (a relative value to 1). These fold changes were calculated using the 2^−ΔΔ*C*^_T_ method^73^. For drawing histograms, all normalizer and fold change values were log2 transformed, and those transformed values were shown in the histograms.

### DNA microarray analysis

The integrity of purified RNAs was assessed using the Agilent 2100 BioAnalyzer (Agilent Technologies, Palo Alto, CA). All samples were confirmed to have an RNA integrity number (RIN) greater than 7. Contaminant DNA was removed by digestion with RNase-free DNase (Qiagen). Using 200 ng of total RNA, complementary RNA was prepared using a one-cycle target labeling and control reagents kit (Affymetrix, Santa Clara, CA). Hybridization and signal detection of HG-U133 Plus 2.0 arrays (Affymetrix) were performed according to the manufacturer’s instructions. Gene expression analysis was performed in triplicate for both monolayer- and Matrigel-cultured SUM149 cells.

### Gene expression data analysis

Data obtained from the six microarrays were normalized by the robust multiarray average (RMA) method using R and BioConductor software^74,75^. Before statistical analysis, we excluded 62 control probe sets and 13,887 probe sets that did not assign any unique gene symbols according to NetAffx Annotation Update Release 30 (http://www.affymetrix.com/analysis/index.affx). We next performed a detection call using Gene Expression Console (Affymetrix) and selected 15,979 probe sets that were marked as “present” in all triplicate assays of either monolayer- or Matrigel-cultured SUM149 cells.

We performed a class comparison with the *t*-test for each gene to identify genes that were differentially expressed between monolayer- or Matrigel-cultured SUM149 cells. To adjust for the multiple comparisons, we calculated false discovery rates and also assessed global significance using BRB-ArrayTools software as previously described^40^. False discovery rates were calculated with the Significance Analysis of Microarrays (SAM) tool as the median number of false-positive genes from permutation testing divided by the number of nominally significant genes defined from the unperturbed data^76^. The global *p* value is the probability of detecting at least the same number of genes of significance on a parametric test (e.g., *t*-test) at the specified *p* level by chance if there are no real differences between monolayer- or Matrigel-cultured SUM149 cells. Probe sets that satisfied both a *p* value of <0.01 and a fold change value of >1.5 for both upregulated and downregulated genes were selected and defined as the “Matrigel gene signature”.

### Gene set enrichment analysis

We conducted gene set enrichment analysis (GSEA) with BRB-ArrayTools software using gene set category C5 from the Gene Ontology database^26^. We used the Efron and Tibshirani gene set analysis method to determine whether gene sets were differentially expressed between monolayer and Matrigel classes, with statistical significance being determined by a permutation test^27^. Gene sets satisfying a *p* value of <0.05 were considered statistically significant. We also performed gene set analysis with five specific gene sets: EMT^28–30^, claudin-low (CL)^31^, immune kinase (IKS)^32^, mitosis kinase (MKS)^32^, and genomic grade index (GGI)^33^. Significantly differentially expressed genes were also functionally interpreted using Ingenuity Pathway Analysis (IPA) software as we have done previously^35^.

### Correlating Matrigel gene signature with gene expression of human breast cancer

Patient tissue sample microarray data were used for comparison with our *in vitro* results. The HG-U133 microarray used for clinical breast cancer samples was different from the HG-U133 Plus 2.0 microarray used for the samples derived from monolayer- or Matrigel-cultured SUM149 cells. Therefore, in order to select the probe sets in HG-U133 that matched those of HG-U133 Plus 2.0, we set the following criteria for the HG-U133 microarray: 1) if a probe set in the Matrigel gene signature identified with HG-U133 Plus 2.0 was also included in HG-U133A, the identical probe set was used; 2) if the selected probe set was not included in HG-U133A, we chose the probe that identified the same gene transcript with the highest average expression in the clinical data sets instead; 3) if no probe set satisfying the first two criteria was found, such genes were discarded for further analysis. Two public data sets, GSE2034 and GSE16716, were downloaded from the GEO database^39,40^. For each microarray data set, we performed hierarchical clustering analysis using the overexpressed genes in the Matrigel gene signature to identify the genes that were overexpressed in both TNBC patient samples and Matrigel-cultured SUM149 cells^35^. Pearson’s correlation coefficient was used to calculate a similarity matrix among genes or patients. The complete linkage method was used for clustering.

### Statistical analysis

A non-paired Student *t*-test was conducted by using the BRB-ArrayTools or R software. In the survival analysis, Kaplan-Meier curves were drawn by R software, and log rank tests were performed to compare the results. *P* < 0.05 was considered statistically significant.

### Data availability

Data matrix file (.txt) and CEL files that we used in microarray data analysis is available in the GEO with the accession number of GSE109396. Other datasets generated in the present study are available from the corresponding authors on reasonable request.

## Electronic supplementary material

Supplementary figures

Supplementary Table 1

Supplementary Table 2

Supplementary Table 3

Supplementary Table 4
