# Supplementary material for: CSF-1/CSF-1R axis is associated with epithelial/mesenchymal hybrid phenotype in epithelial-like inflammatory breast cancer
Source: Sci Rep. 2018 Jun 21;8:9427. doi: 10.1038/s41598-018-27409-x (PMC6013474; doi:10.1038/s41598-018-27409-x)
Supplement: Supplementary file 1 — Supplementary figures [file 41598_2018_27409_MOESM1_ESM.pdf]

CSF-1/CSF-1R axis is associated with epithelial/mesenchymal hybrid phenotype in epithelial-like inflammatory breast cancer

Kazuharu Kai[1,2,3], Takayuki Iwamoto[6], Dongwei Zhang[2,3], Li Shen[4], Yuko Takahashi[6], Arvind Rao[4], Alastair Thompson[5], Subrata Sen[1,*] and Naoto T. Ueno[2,3,*]

[1]Department of Translational Molecular Pathology, [2]Department of Breast Medical Oncology, [3]Morgan Welch Inflammatory Breast Cancer Research Program and Clinic, [4]Department of Bioinformatics and Computational Biology and [5]Department of Breast Surgical Oncology, The University of Texas MD Anderson Cancer Center, Houston, Texas. [6]Department of Breast and Endocrine Surgery, Okayama University Hospital, Okayama, Japan.

***Corresponding authors:**

Subrata Sen, Ph.D. (ssen@mdanderson.org). Phone: (713) 834-6040; Fax: (713) 745-8221

Naoto T. Ueno, M.D., Ph.D., F.A.C.P. (nueno@mdanderson.org). Phone: (713) 792-8754; Fax: (713) 794-4385

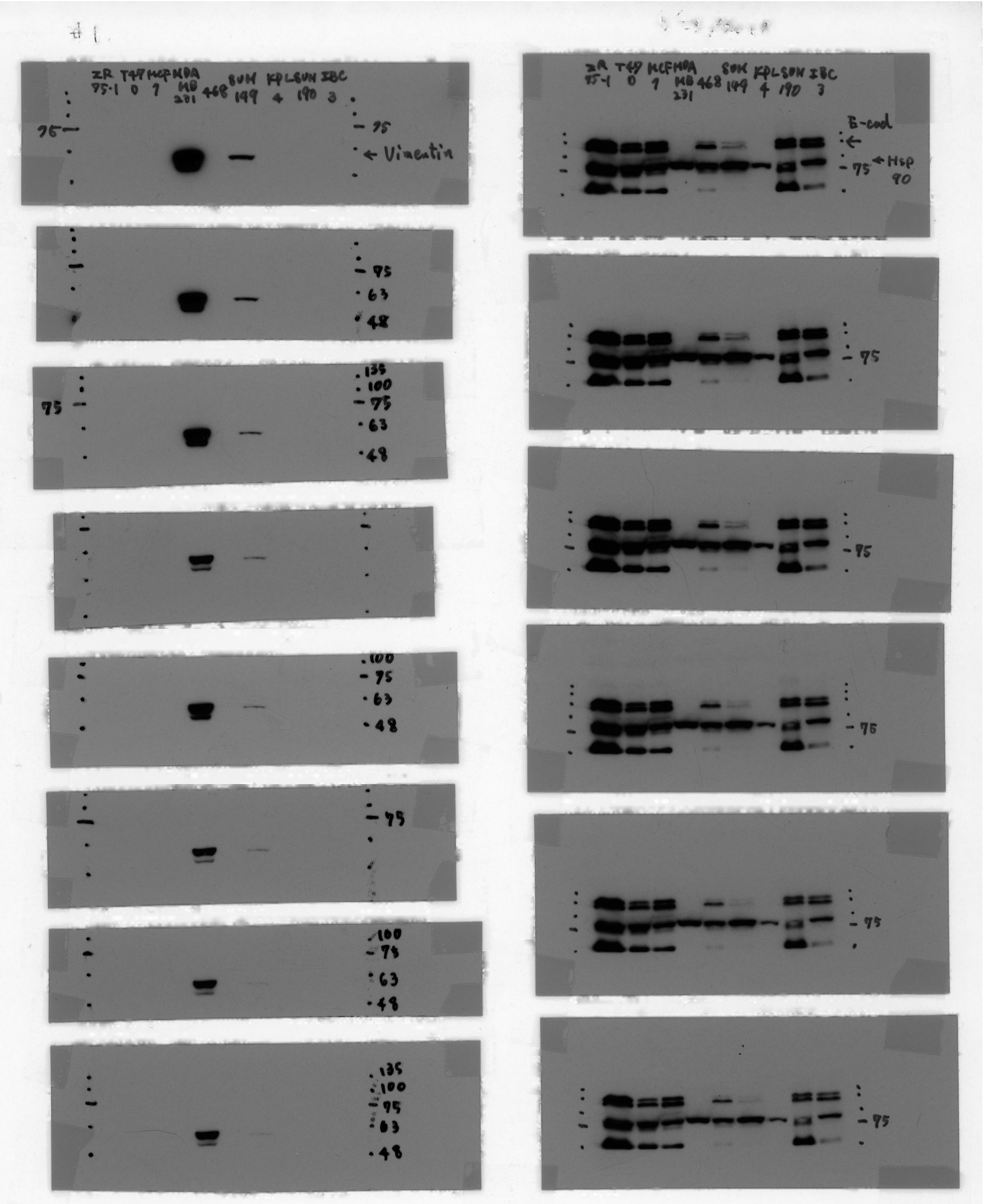

Supplementary Figure S1. Original blot images for Vimentin (left) and E-cadherin (right) used for Figure 1. Images are ordered according to the exposure time: long exposure (top) to short (bottom). In the right blots, E-cadherin (molecular weight: 135 kDa) and Hsp90 (molecular weight: 90 kDa) were detected with the same blot.

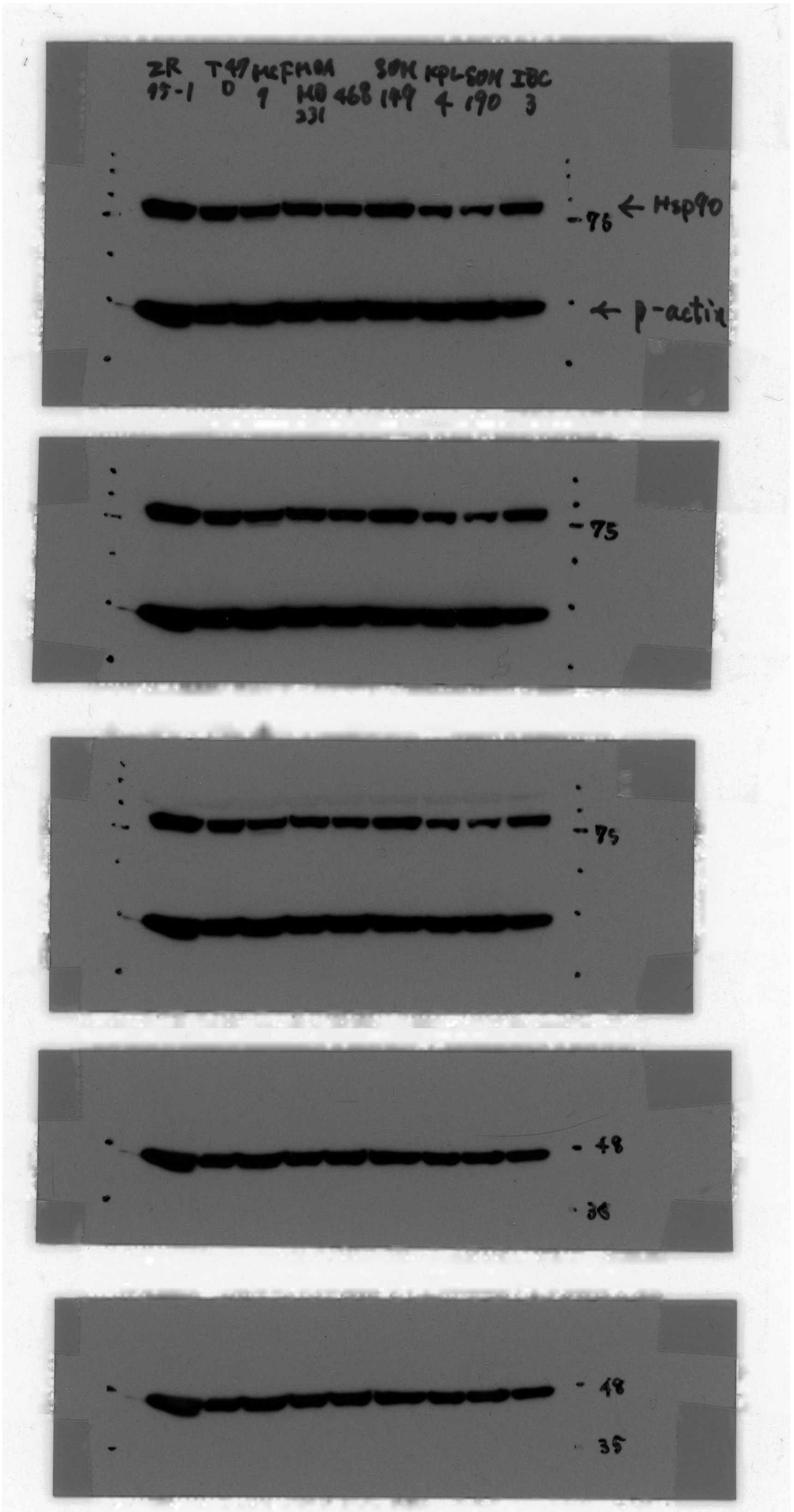

Supplementary Figure S2. Original blot images for β-actin used for Figure 1A. Images were taken after stripping antibodies agaist Vimentin and E-cadherin followed by probing with antibodies agansit β-actin alone (lower 2 blots) or β-actin and Hsp90 (upper 3 blots). Uppler 3 images are derived from the same membrane shown in the left of Supplementary Figure S1. Lower 2 images are derived from the same but a lower part of membrane shown in the right of Supplementary Figure S1.

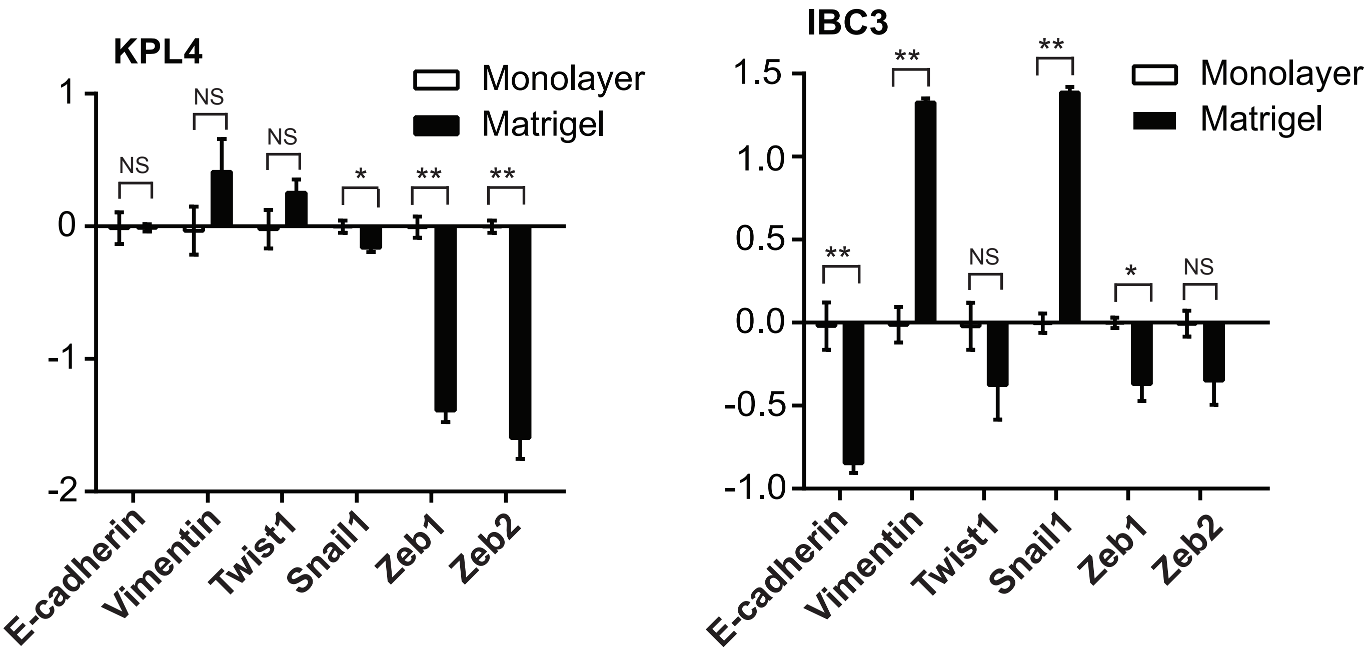

Supplementary Figure S3. Transcriptional changes in EMT makers induced by Matrigel culture in KPL4 and IBC3 inflammatory breast cancer cells. Transcriptional profiling of EMT markers was performed by quantitative RT-PCR with KPL4 and IBC3 inflammatory breast cancer cells cultured in monolayer or Matrigel culture conditions. For each marker, samples from monolayer culture condition were set as a normalizer and all relative expression values were log2 transformed. Bars, standard error of mean.

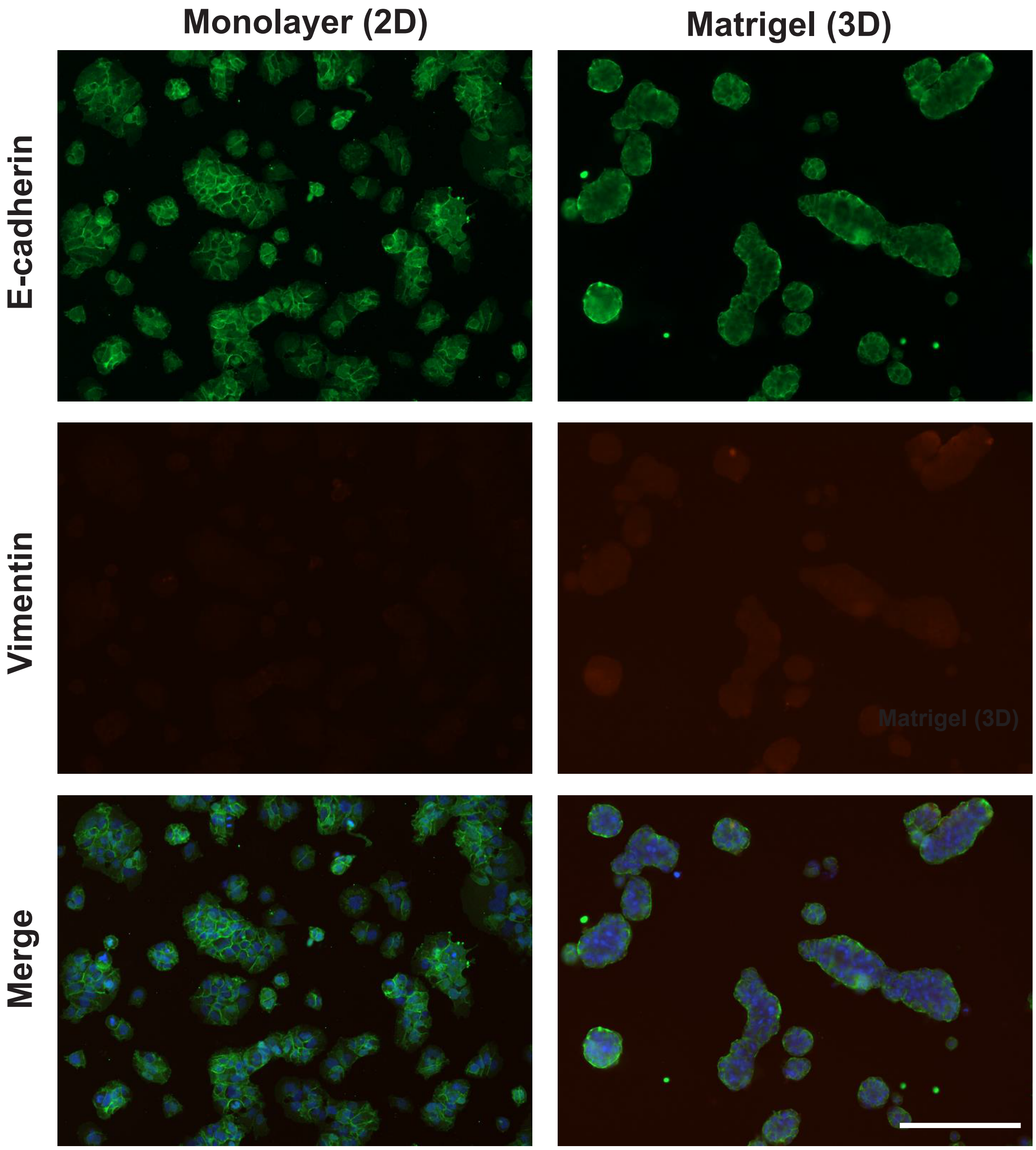

Supplementary Figure S4. Immunofluorescent analysis for E-cadherin and vimentin in SUM190 cells cultured in monolayer or Matrigel. Merged images are derived from the images of E-cadherin, vimentin, as well as nuclear counterstaining DAPI (Blue). Bar, 200 μm.

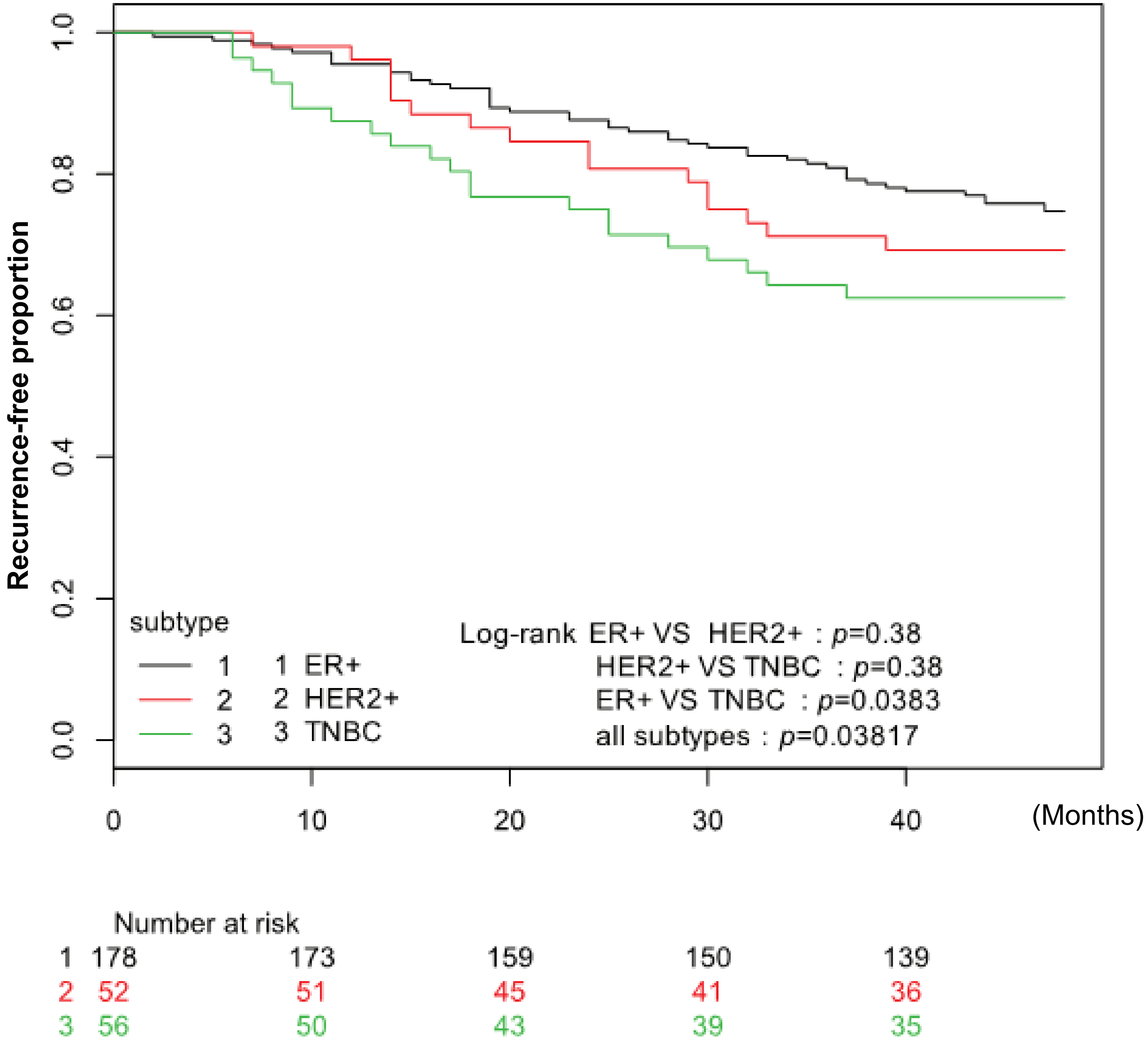

Supplementary Figure S5. Kaplan-Meier curve for recurrence free survival in primary breast cancers (Wang *et al*, Lancet 2005) according to molecular subtypes. Logrank tests were conducted for the comparisions between two sutypes and for all three subtypes.

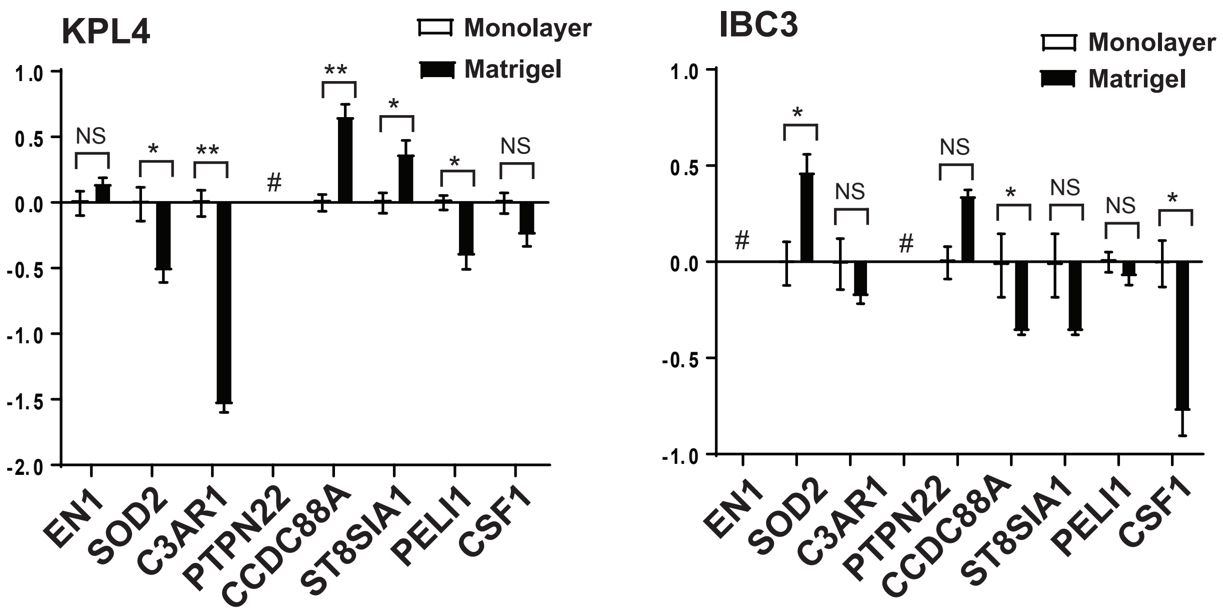

Supplementary Figure S6. Transcriptional changes in inflammatory-related genes induced by Matrigel culture in KPL4 and IBC3 inflammatory breast cancer cells. Transcriptional profiling of inflammatory-related genes was performed by quantitative RT-PCR with KPL4 and IBC3 inflammatory breast cancer cells cultured in monolayer or Matrigel culture conditions. For each gene, samples from monolayer culture condition were set as a normalizer and all relative expression values were log2 transformed. Bars, standard error of mean.

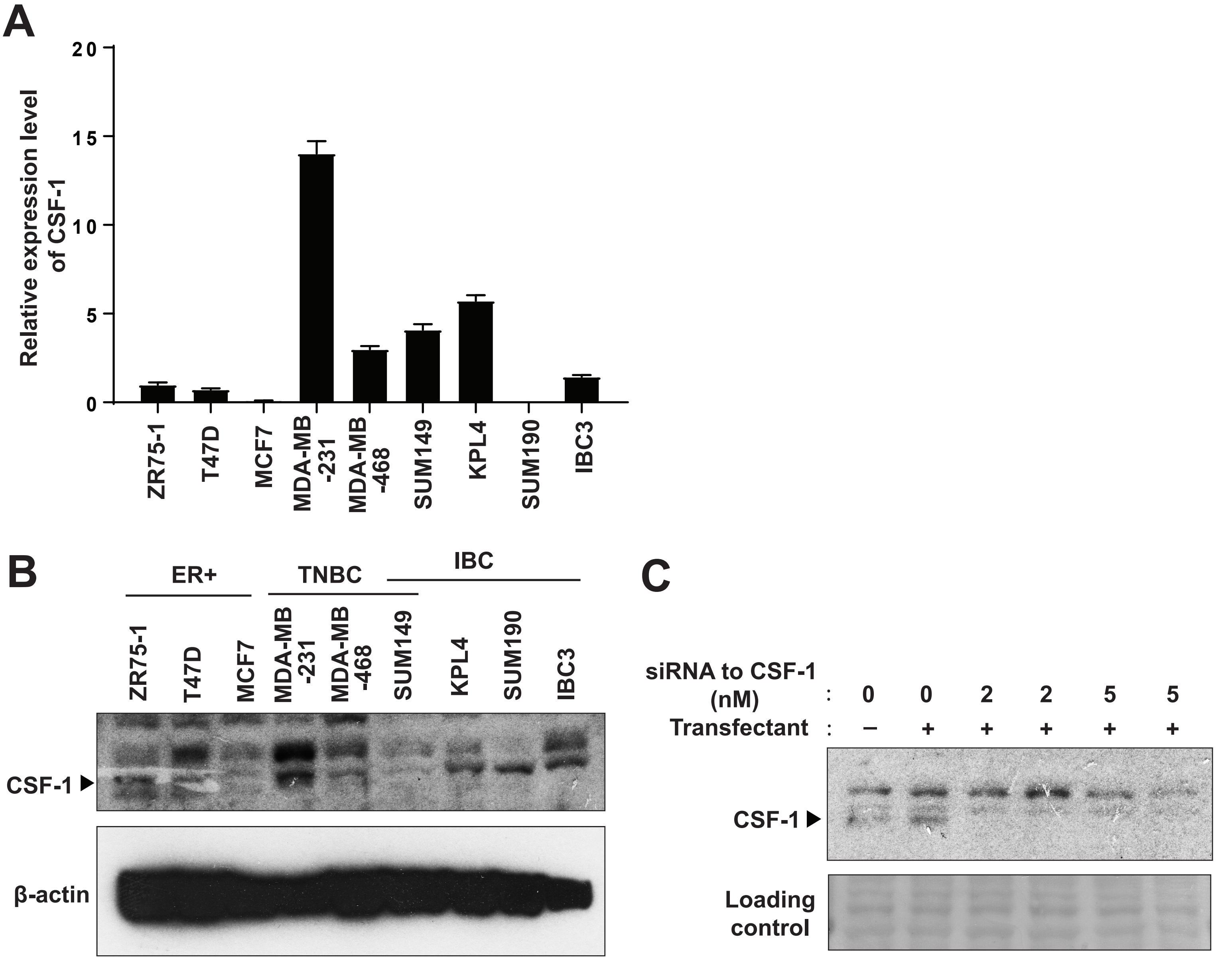

Supplementary Figure S7. Transcriptional and protein expressions of CSF-1 in a panel of breast cancer cells. A, transcriptional expressions of CSF-1 determined by TaqMan RT-PCR. ZR75-1 is a normalizer (set as the expression value=1) and other cell lines' values are relative values to the ZR-75-1's. The values are log2 transformed. **B,** protein expressions of CSF-1 detected by western blots. Arrowhead indicates the band that corresponds to CSF-1 (defined by **C**). β-actin, loading control. **C**, Knocking down of CSF-1 with siRNA to CSF-1 in MDA-MB-231 cells, which confirmed the band corresponding to CSF-1.

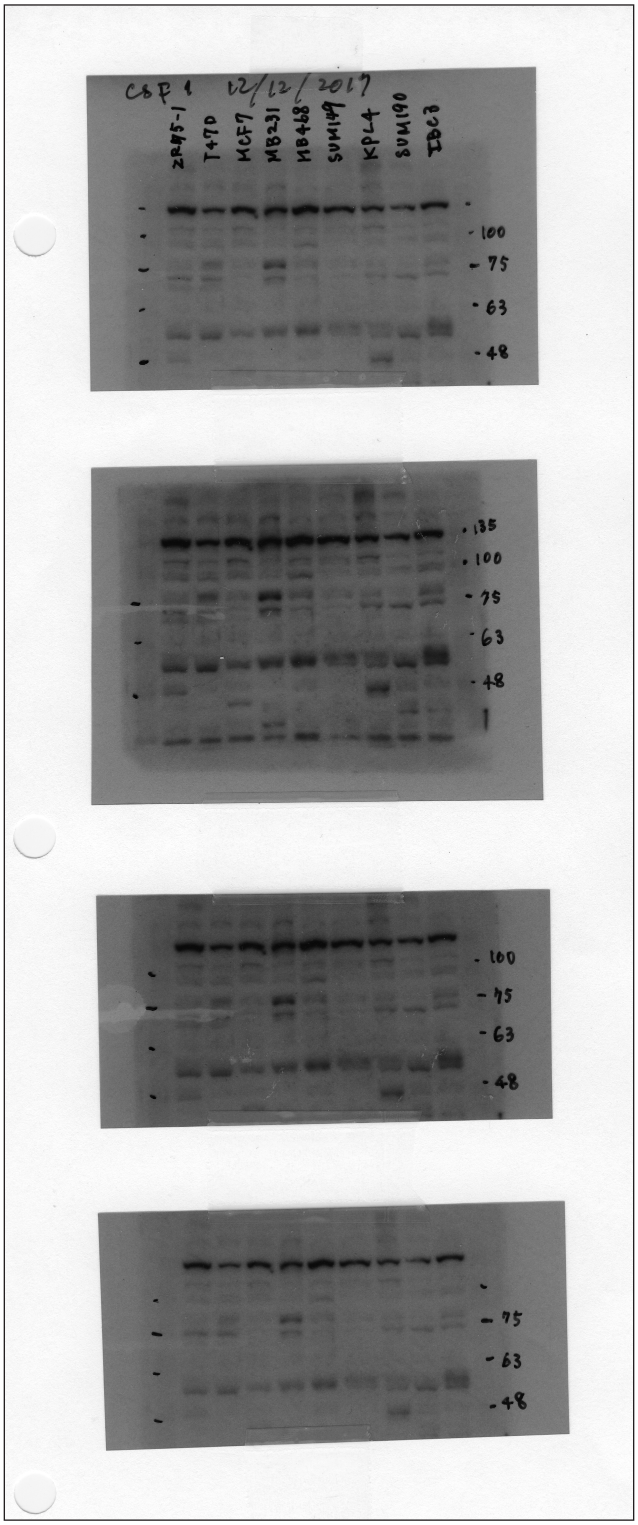

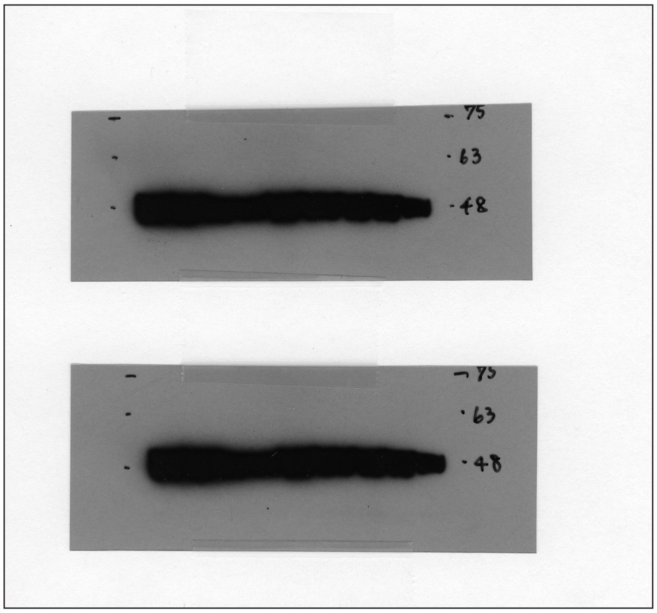

Supplementary Figure S8. Original blot images for CSF-1 (left) and β-actin (right) used for Supplementary Fig. S7.

Membrane was first probled with anti-CSF-1 antibody and detected CSF-1 (Left, molecular weight: around 70 kDa). After this detection with different exposure times, the same membrane was probed with anti-β-actin antibody and detected β-actin (Right, molecular weight: 42 kDa).

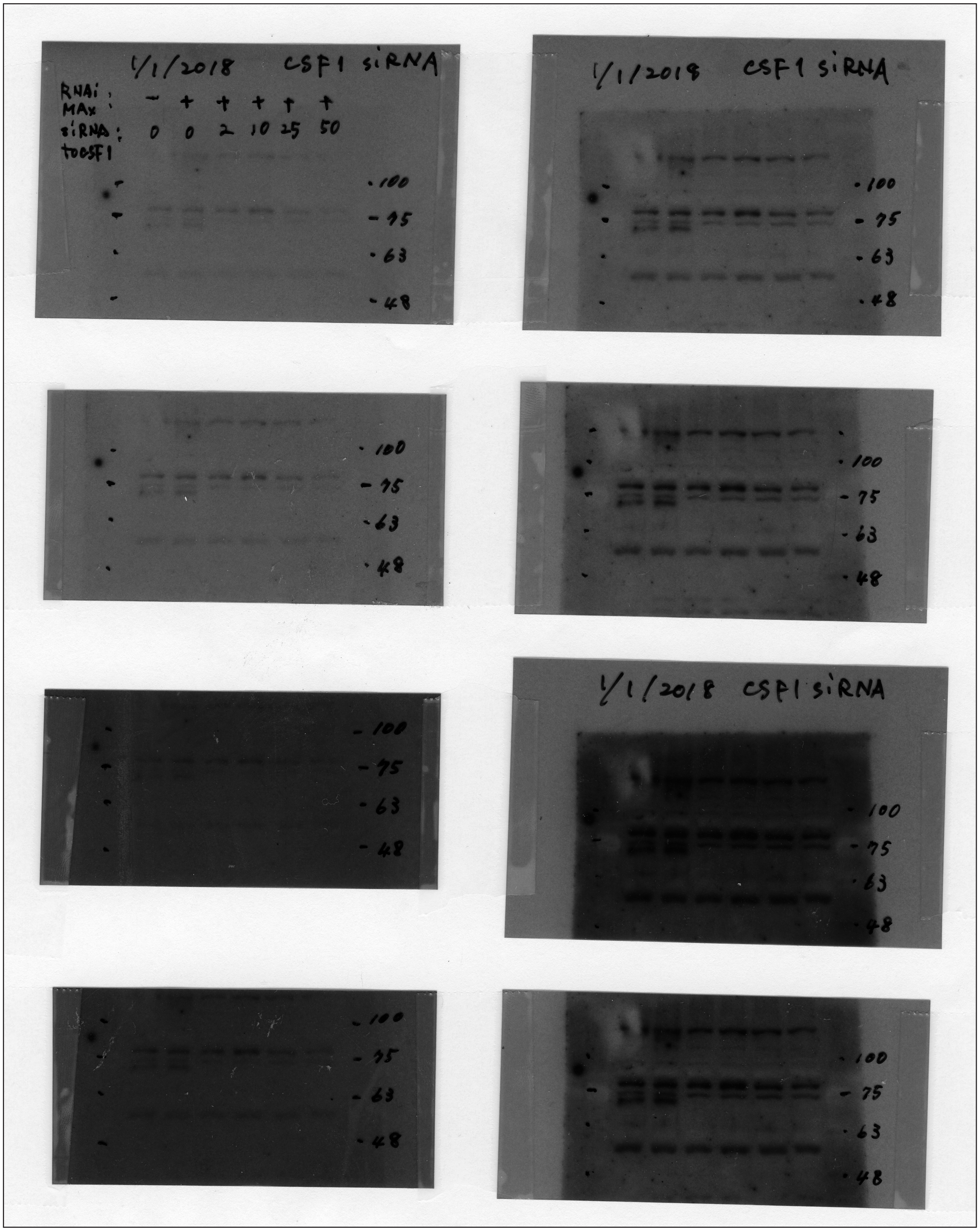

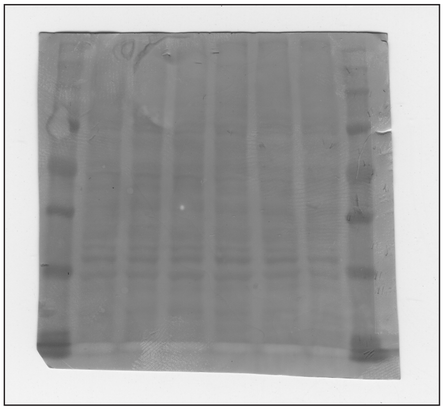

Supplementary Figure S9. Original blot images for CSF-1 (upper) and the same membrane image after staining with Ponceau S used for Supplementary Fig. S7.
Membrane was first probled with anti-CSF-1 antibody and detected CSF-1 (Uppler, molecular weight: around 70 kDa). After this detection with different exposure times, the same membrane was processed for Ponceau S staining (Lower).

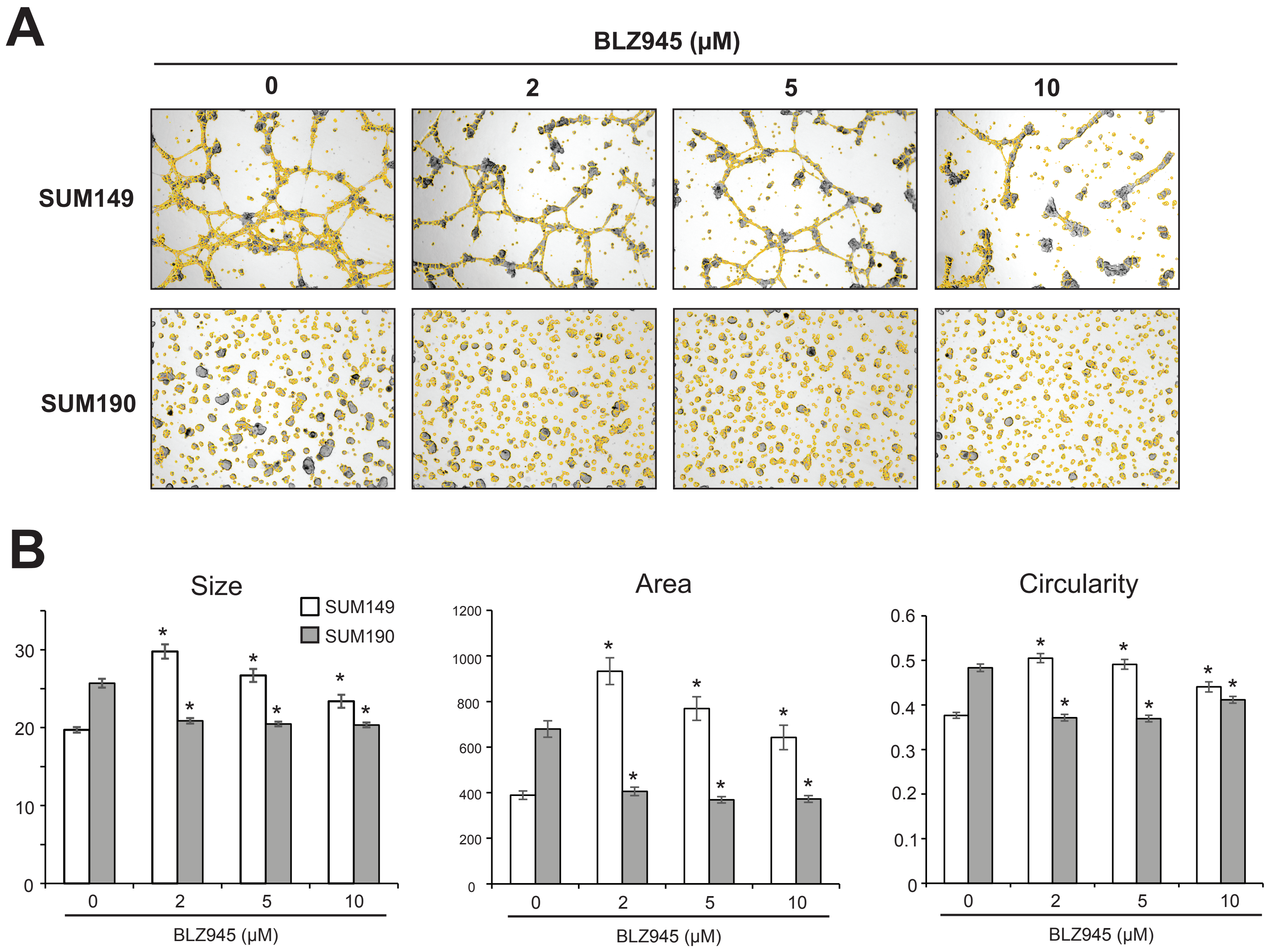

Supplementary Figure S10. Effect of CSF-1R inhibitor BLZ945 on the morphology of IBC cells cultured in Matrigel. A, Images shown in Fig. 4B were analyzied with a function of Gen5 software, Cellular Analysis, which automatically delineates the bounderies of single cells or small cell clumps and quantifies cell size, area, and circularity. **B,** Histograms depict average cell size, area, and circularity of Matrigel-cultured SUM149 and SUM190 cells treated with different concentrations of BLZ945. Every concentrations of BLZ945 induced statistically significant morphological changes compared to non-treatment in every measures; size, area, circularity, in both SUM149 and SUM190 cells. *, $p<0.05$ in a comparion with the cell line-matched non-treated control.

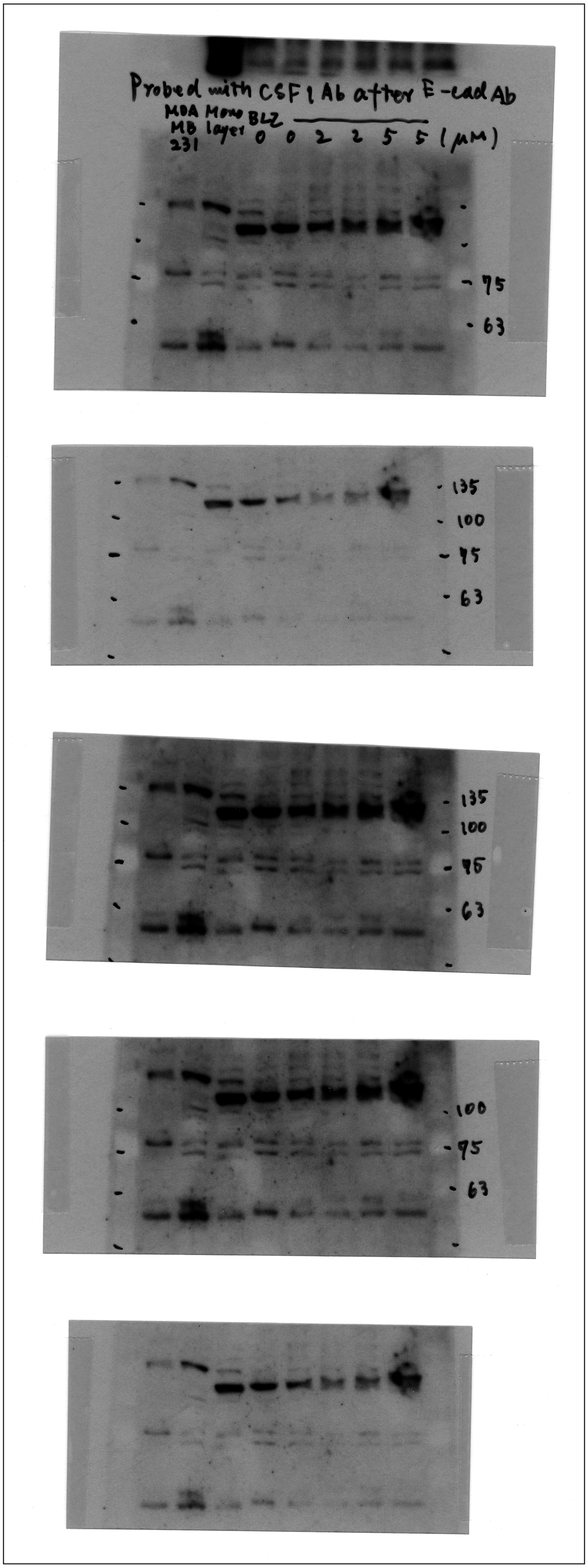

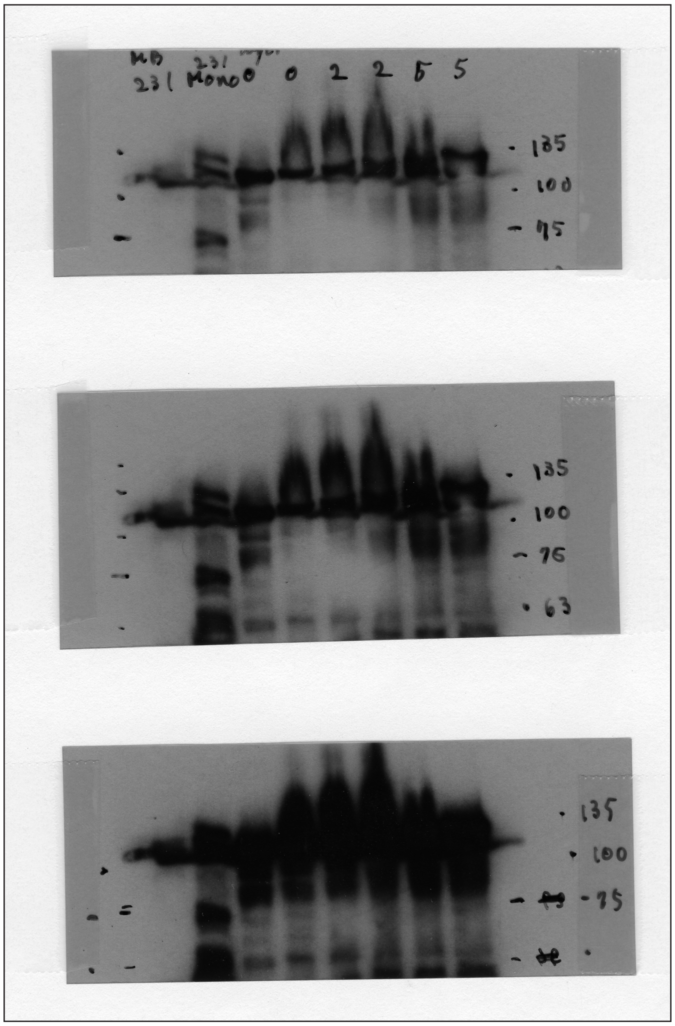

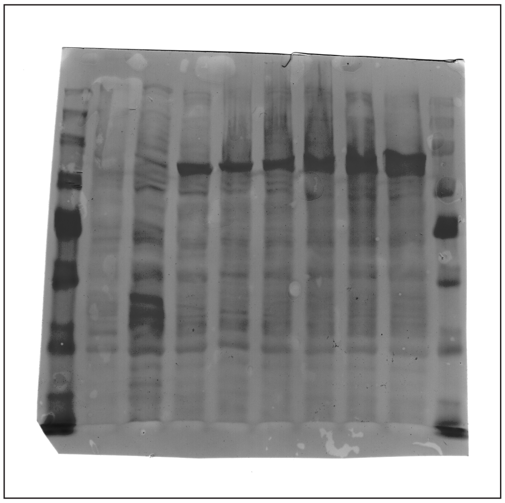

Supplementary Figure S11. Original blot images for CSF-1 (left), E-cadherin (uppe right) and the same membrane image after staining with Ponceau S used for Fig. 4D.
Membrane was first probled with anti-E-cadherin antibody and detected (Right upper, E-cadherin molecular weight: 135 kDa), followed by the detection of CSF-1 (Left, molecular weight: around 70 kDa). After these detections with different exposure times, the same membrane was processed for Ponceau S staining (Right lower).
